# Injury risk and running habits in parents: Does stroller use make a difference?

**DOI:** 10.1371/journal.pone.0354371

**Published:** 2026-08-12

**Authors:** Houn G. Kim, Andrew Friesen, Allison R. Altman-Singles

**Affiliations:** 1 Department of Biology, Pennsylvania State University, Reading, Pennsylvania, United States of America; 2 Department of Kinesiology, Pennsylvania State University, Reading, Pennsylvania, United States of America; 3 Department of Mechanical Engineering, Pennsylvania State University, Reading, Pennsylvania, United States of America; Geisinger Health System, UNITED STATES OF AMERICA

## Abstract

Running with a stroller allows parents to stay physically active while looking after their children. However, the impact of stroller running on overuse injury risk among parents of young children is not well understood. We hypothesize that stroller runners may experience fewer injuries than non-stroller runners, due to differences in running mechanics and impact load as informed by prior biomechanical literature. This study seeks to characterize running habits and injury patterns in parent runners, examining whether stroller use affects running-related injury incidence and mileage. Parent runners were recruited in this cross-sectional study via email and social media platforms to complete a web-based survey, self-reporting data on demographics, weekly running volume, and injury history. Two groups were identified in this study: stroller (n = 200; parents who chose to run with a stroller) and control (n = 55; parents who chose not to run with a stroller). Statistical analyses were conducted to compare running mileage and injury incidence in the first three years of parenting between groups. The final analytic sample for injury analyses comprised 196 participants in the stroller group and 53 in the control. Overuse injuries were more prevalent among non-stroller runners (30%) than in stroller runners (19%), with the stroller group experiencing a lower injury rate (0.19 per 1,000 miles) compared to the control (0.42 injuries per 1,000 miles). Injury type and distribution were similar between groups. There was a notably higher incidence of plantar fasciitis reported among both stroller and non-stroller runners. Stroller running appears to be a generally safe and accessible form of exercise for parent runners, with lower injury incidence observed compared to non-stroller runners. Findings in this study underscore that running with a stroller may support sustained physical activity during early parenthood.

## Introduction

Running is a popular form of physical activity that offers a range of physical and mental health benefits [1,2]. It serves as an accessible, low-cost activity to engage in regular exercise for millions of individuals [1,3]. Among runners, parents represent a unique sub-population who often seek innovative ways to balance the demands of caregiving with maintaining an active lifestyle. For many, running with a stroller has become a practical means to integrate exercise into daily routines, enabling parents to remain physically active while caring for their children [3,4].

Running-related injuries are multifactorial, with research indicating that 19–79% of runners are injured each year [5]. This high incidence is driven by several key potential risk factors that contribute to the onset of overuse injuries, including gait mechanics, training patterns, hormonal changes, energy availability, and a history of prior injuries. Injury type and distribution can vary by sex, with females being more susceptible to knee pain and stress fractures, while males are more prone to foot and ankle injuries [6–8]. The most prevalent injuries include patellofemoral pain syndrome, iliotibial band syndrome, achilles tendinopathy, medial tibial stress syndrome, and stress fractures [5]. Epidemiological studies indicate that knee injuries account for approximately 25%, making it the most common site for running overuse injury [9,10]. Overall, about 70–80% of running injuries occur from the knee downward [9,10]; thus, it is important to consider how the altered mechanics of stroller running may influence the distribution and frequency of these injuries among parent runners.

Previous investigations have consistently found that abnormal lower extremity kinematics – such as excessive dynamic foot pronation [11] or greater peak hip adduction and knee internal rotation [12] are significant predictors of running injuries. Notably, high vertical impact loading – characterized by elevated ground reaction forces and rapid load rates – has been identified as a key predictor of overuse injuries, including stress fractures and soft tissue damage [12]. Studies show that runners with higher impact loading are more likely to sustain injuries than those with lower loading patterns [13]. Moreover, increased torsional loads, which refer to twisting forces experienced during running, have also been shown to elevate the risk of overuse injuries, particularly when combined with high impact forces [14]. Previous work from Mahoney and colleagues demonstrated that running with a stroller significantly reduces impact loading, while increasing torsional loading [15]. Consequently, stroller use may affect the injury risk profile of parent runners.

While many parents choose to run with strollers after having children, the specific relationship between the biomechanical changes induced by stroller running and injury risk during early parenthood remains unclear. Literature indicates that running with a stroller often results in shorter stride length, slower running speed, and increased ratings of perceived exertion, suggesting that the task is more physically demanding [4]. Additionally, while the altered running mechanics-such as increased trunk lean, reduced trunk rotation, and increases in anterior pelvic tilt and hip flexion [16] – may theoretically increase the risk of overuse injuries, current evidence does not conclusively link stroller running to a higher incidence of injury compared to the general running population. Since stroller running considerably modifies running mechanics, it is crucial to assess whether these biomechanical adaptations mitigate or exacerbate injury risk in parent runners.

In addition to gait-related risk factors of overuse injury, training habits – particularly increased mileage and intensity – are closely associated with injury risk. Several studies demonstrated that runners who increase their weekly running distance or training intensity too rapidly are at greater risk of injury [17,18]. For instance, research has shown that sudden increases in weekly running distance by 20–60% can significantly elevate injury risk in the short term, and that novice runners who progress their running distance by more than 30% over a two-week period are especially vulnerable to distance-related injuries [18]. Training errors – such as running too frequently, at too high an intensity, or for excessive durations – are also major contributors to injuries in both novice and experienced runners [19,20]. Runners who prioritize intensity over volume face a 15% higher risk of injury compared to those who emphasize distance [21]. Additionally, a running schedule centered on intensity is more likely to lead to injuries such as achilles tendinopathy, gastrocnemius strains, and plantar fasciitis, rather than distance-related injuries [22]. Given that parent runners may adjust their running volume or intensity due to the demands of caring for their children, this shift in training habits could influence their susceptibility to injury.

Furthermore, studies have found a low but significant correlation between irregular training habits and running injuries [9]. Research has shown that novice runners, who often run irregularly, have a significantly higher incidence of injuries – up to 17.8 per 1000 hours – compared to more consistent recreational runners, who experience about 7.7 injuries per 1000 hours [22]. In addition, abrupt changes in training habits can lead to running injuries as the muscular tissues may lack the capacity to adapt to sudden stresses [23]. A sudden change to a specific form of training – such as interval or hill training – without a gradual buildup is also considered a training error [24,25]. Studies have indicated that abrupt changes in training routines have been associated with 60% of all running injuries [26]. For parents, the unpredictable demands of childcare may contribute to more irregular running habits, which could increase injury risk when running with a stroller.

Epidemiological research has also suggested that individuals with a previous injury may be at greater risk of being reinjured due to persistent underlying causes, reduced functionality or protection of the healed tissue, or incomplete recovery [23]. Study analysis reported that runners with a history of previous injury had a 65% higher risk of sustaining another injury, even after accounting for differences in weekly running distance [27]. Therefore, the physical stresses of stroller running may compound the effects of a previous injury, potentially affecting the overall risk in parent runners of sustaining another running-related injury. Additionally, research highlighted that runners who incorporated cross-training may experience fewer injuries compared to those who only run [28]. This is likely due to corrections in strength imbalances by conditioning key muscles not impacted by running as well as substitutions of non-weight-bearing activities like swimming or cycling for part of the weekly running mileage, thereby reducing impact-related stress [28]. These findings indicate that incorporating cross-training and varied methods of loading into a running routine may help parent runners minimize injury risk while maintaining overall fitness.

Additional studies have also demonstrated that hormonal fluctuations significantly influence injury risk in runners. Specifically, pregnancy, marked by elevated levels of estrogen and progesterone, is believed to increase connective tissue compliance, which may contribute to a heightened risk of sustaining an injury while running [29]. Moreover, research has shown that a decrease in connective tissue stiffness is linked to reduced power output and higher risk of ligament injuries [30]. Furthermore, studies revealed that running is associated with a concomitant decline in testosterone levels and a 4.5-fold increase in the likelihood of sustaining bone injuries [31,32]. Hence, these hormonal factors should also be considered when evaluating the impact of stroller use on injury patterns among parent runners.

Consequently, this study ultimately aims to address the gap in our understanding of how these varying factors influence running habits, injury incidence, and the overall running experience among parent runners. By characterizing the running habits and distribution of injuries in parents of young children who run with a stroller, this research seeks to explore how stroller use may affect injury risk in this sub-population. It was hypothesized that stroller runners may sustain fewer injuries compared to non-stroller runners. This hypothesis is based on previous biomechanical literature that identified reduced impact load when running with a stroller [15].

## Methods

Parents of young children who chose to run with or without a stroller were recruited from May 23, 2024 until November 25, 2025, to complete a web-based survey (Qualtrics, Provo, UT, USA), designed to assess running habits and injury risks. The cross-sectional study protocol was reviewed and determined exempt from review by the authors’ Institutional Review Board (IRB), based on minimal risk and interaction with study participants. At the beginning of every survey, individuals were presented with an informed consent statement indicating that the following survey was for research purposes and that they could discontinue their participation at any time. Once participants checked a box indicating “yes” they understand they are consenting to participate in this research, they were then directed to the eligibility screening questions. This method of informed consent was approved as exempt by the Penn State Institutional Review Board (STUDY00024506).

An *a priori* power analysis determined that 150 total participants would be needed to observe a 30% increase in injured runners relative to the null hypothesis (α = 0.05, β = 0.95). Participants were eligible for the study if they met the following inclusion criteria: aged between 18 and 55 years; had been a parent to children aged 0–3 years within the last 20 years; had been a parent for a minimum of three years; ran more than 5 miles per week at some point before becoming a parent; and were medically able to run during the period when their child was between six months and three years of age. Respondents who did not meet these criteria were excluded from the study. Individuals who qualified for the study were asked to detail their running injuries during the period of time stroller use was most common, when their children were aged 0–3 years.

Potential subjects were recruited electronically via email invitations sent to U.S.-based and international running clubs, as well as through posts on social media platforms, accompanied by a QR code for easy access to the survey. All recruitment materials included a brief description of the study, eligibility requirements, and a link to the online survey. The survey used in this study was adapted from previous running-related injury research [33,34] and modified for this unique sub-population.

Participants must have resumed running after becoming a parent prior to group allocation. Parents who continued to run after having children were separated into two groups: those who resumed running with a stroller (stroller group) and those who did not use a stroller (control group). Respondents who failed to report whether they continued to run post-parenthood or whether they ran with or without a stroller were removed from the final analytic sample. The survey included questions regarding self-reported data on demographics (e.g., age, height, weight, biological sex, and number of children), running volume (e.g., weekly mileage between 0–10 years postpartum), and history of overuse injuries (e.g., injury type, anatomical location of injury, and injury duration). Overuse injuries were considered any injury that were self-reported by participants regardless of clinical treatment, pain, duration, or impact on training.

Descriptive and inferential statistical analyses were conducted to examine differences in running volume and injury prevalence between stroller and non-stroller runners. This study was a between-subjects design comparing between stroller and control groups, where the primary independent variables were injury incidences and injury rate. Prior to inferential analyses, assumptions for parametric testing were evaluated to determine whether the data met the criteria required for valid use of t-tests when comparing between the stroller and control groups. Normality of continuous variables (mileage and injury rate) were assessed using Shapiro-Wilk test, and homogeneity of variance was analyzed using Leven’s test to establish whether equal variance assumptions were met. When assumptions for parametric testing were not met, secondary non-parametric analyses were performed. Kolmogorov-Smirnov tests were also conducted to confirm distribution differences in injury rate variables. These non-parametric results were interpreted using an alpha level of *p* < 0.05 (two-tailed).

All data analyses were conducted using Microsoft Excel. Summary data were reported as mean ± SE for continuous variables, such as age, height, weight, age at first child, number of children, and weekly running mileage. Demographic data were also compared between groups using either T-tests or Chi-Square tests, based on the data type. The percentages for categorical variables, including running-related injuries were calculated, and the number of each specific overuse injury type was summarized descriptively in both groups. ANOVA (group x child age) was conducted on mileage data, and when significant main effects were revealed, *post hoc* t-tests were used to compare mileage between groups at different child age stages. Chi-Squared tests were used to compare the proportion of injured runners in each group. Between-group differences in injury rates per 1000 miles were examined using Mann-Whitney U tests, with corresponding significance values and 95% confidence intervals (CI) reported. Statistical significance was set at an alpha level of *p* < 0.05.

## Results

**Fig 1:**
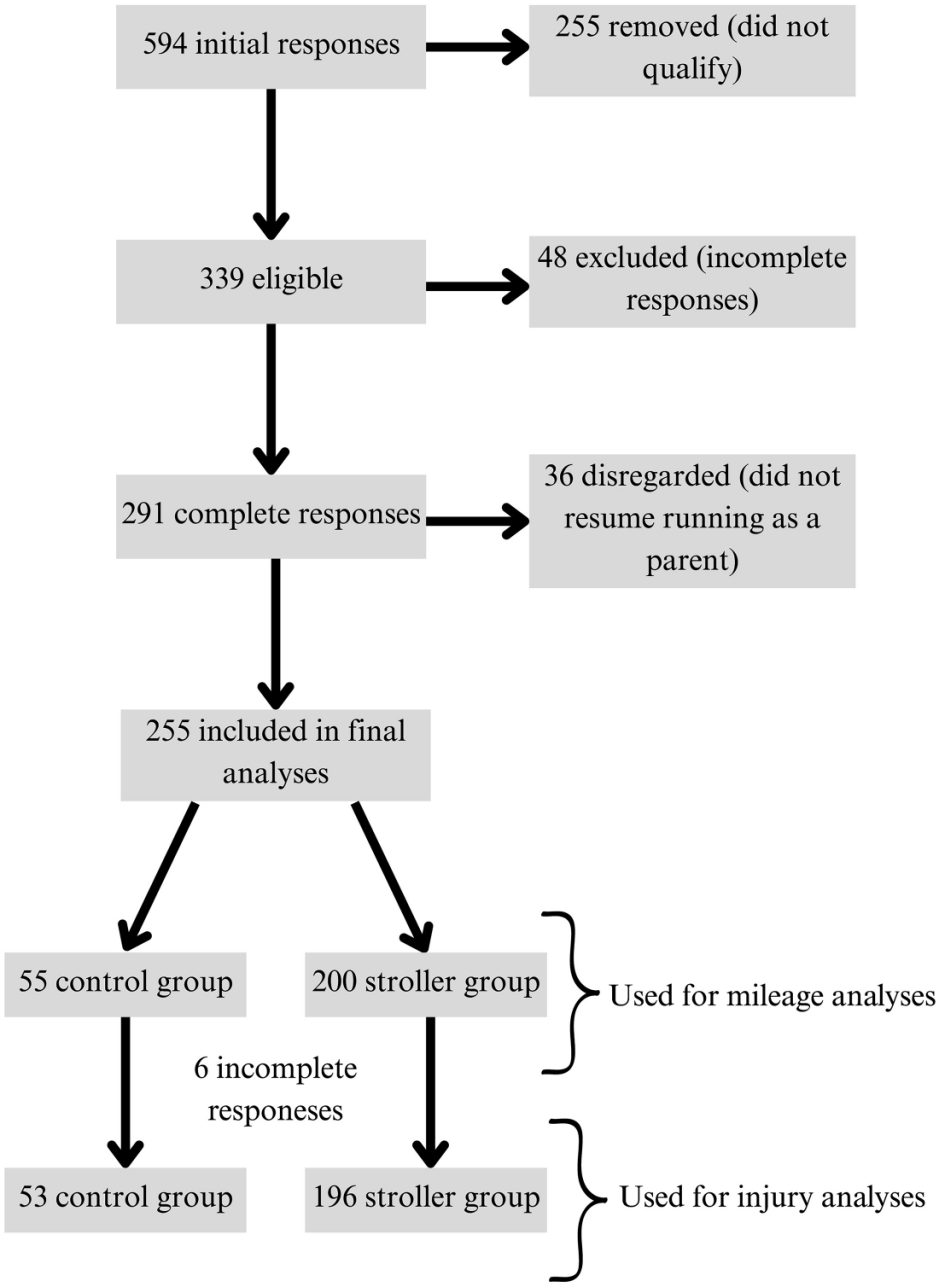
Flow diagram of participant selection for analysis.

A convenience sample of 594 runners attempted the survey (Fig 1). Of these, 255 were removed for not meeting the study’s inclusion criteria, determined *a priori*, leaving 339 participants. After collection of the survey was complete, 48 additional participants were excluded due to incomplete responses, specifically for not indicating whether they continued running after becoming a parent or whether they chose to run with or without a stroller. Of the 291 eligible respondents, 36 did not resume running after becoming a parent and were therefore disregarded from the injury and mileage analyses as these participants could not provide post-parenthood running mileage or injury data relevant to the study objectives. Consequently, 255 runners were included in the mileage analysis, with 200 participants choosing to run with a stroller (stroller), while 55 did not (control). Following the exclusion of six additional participants (control = 2; stroller = 4) due to insufficient data, 249 runners were used for the injury analysis, featuring 196 stroller runners and 53 non-stroller runners.

All female participants in our study reported being the birthing parent (Table 1). Control and stroller groups were not different in age, height, weight, age at first child, and number of children. Both groups were mostly female, but the stroller group was proportionally more female than the control group (*p* < 0.01).

**Table 1:** Demographic profile of stroller and control groups.

Characteristics	Stroller (n = 200)	Control (n = 55)
Age (yrs)	40 ± 0.77	41 ± 0.35
Height (in)	66 ± 0.53	66 ± 0.25
Weight (lbs)	146 ± 3.63	150 ± 1.85
Female*	180 (90%)	40 (73%)
Age at first child (yrs)	31 ± 0.56	30 ± 0.29
Number of children	2 ± 0.15	2 ± 0.05

Numeric data are presented as mean ± SE. Proportions are recorded as number and percentage. * indicates a significant difference between groups (*p* < 0.05).

The most commonly used stroller types among parent runners were single jogging strollers with a locked front wheel (n = 130) and those with an unlocked front wheel (n = 114). Relatively fewer participants reported using double jogging strollers, opting for either models with an unlocked front wheel (n = 60) and a locked front wheel (n = 60). Use of four-wheeled strollers that were not specifically designed for running were used by only nine individuals (n = 7 single non-jogging; n = 2 double non-jogging).

None of the variables, including weekly mileage and injury rate data, were normally distributed based on a Shapiro Wilk test of normality (*p* < 0.001). Injury rate data also violated the assumption of equal variances (Leven’s test, *p* = 0.003; Kolomogrov-Smirnov test, *p* = 0.02), whereas mileage data met the assumption of homogeneity of variance, therefore non-parametric tests were used throughout mileage and injury data.

**Fig 2:**
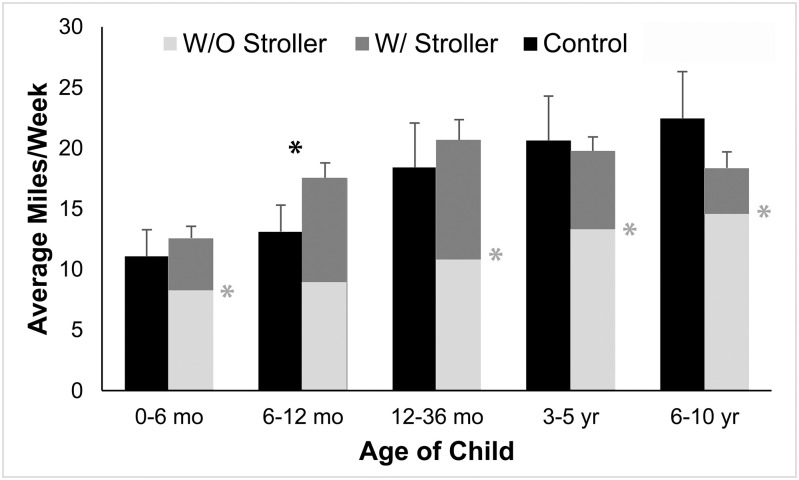
Average weekly running mileage. Weekly mileage in early parenthood when the child is 0 months old through 10 years old for those running with strollers (mileage with strollers – dark gray, mileage without strollers – light gray), and those who do not run with strollers (control – black). Error bars indicate SE. Black * indicates a trend towards a significant difference between groups, and gray * indicates a significant difference between stroller and non-stroller mileage in the stroller group.

There was a significant interaction effect between group and child age stages (*p* = 0.04), with higher running mileage in the stroller group during the earlier stages, but lower mileage as their child reached 3–10 years of age. *Post hoc* analyses revealed that the total miles run by the stroller group (including with and without strollers) and the control group were not different, except for when their children were between 6–12 months old (Fig 2). During this period, stroller runners had a 34% higher net mileage than the control (*p* = 0.015). Overall, weekly mileage varied among participants across all age stages. Weekly mileage was lowest in both groups when the child was 0–6 months, and steadily increased to a plateau between 12 months and 10 years.

Within the stroller group, running with a stroller accounted for 34% of their total mileage when their child was aged 0–6 months (Fig 2). When mileage was paired within individuals, the Wilcoxon signed-rank test revealed the stroller group consistently ran more miles without a stroller than with one when the child was 0–6 months old (*p* < 0.001). When the child was 6–12 months, stroller runners ran 49% of their mileage with a stroller, which was not different from the amount of miles ran without a stroller (*p* = 0.246). By the time the child was 12–36 months old, mileage shifted back towards running without a stroller (*p* = 0.04), with 48% of their mileage run with a stroller. Between ages 3–5 years, stroller running made up 33% of total mileage (*p* < 0.001), and decreased to 21% when the child was aged 6–10 years (*p* < 0.001). Weekly stroller mileage increased in the stroller group from 0–36 months of age, reaching a peak in the period where the child was 12–36 months, and subsequently declined as the child got older. It was notable that 138 runners continued to use strollers until their children were between 6–10 years of age.

**Fig 3:**
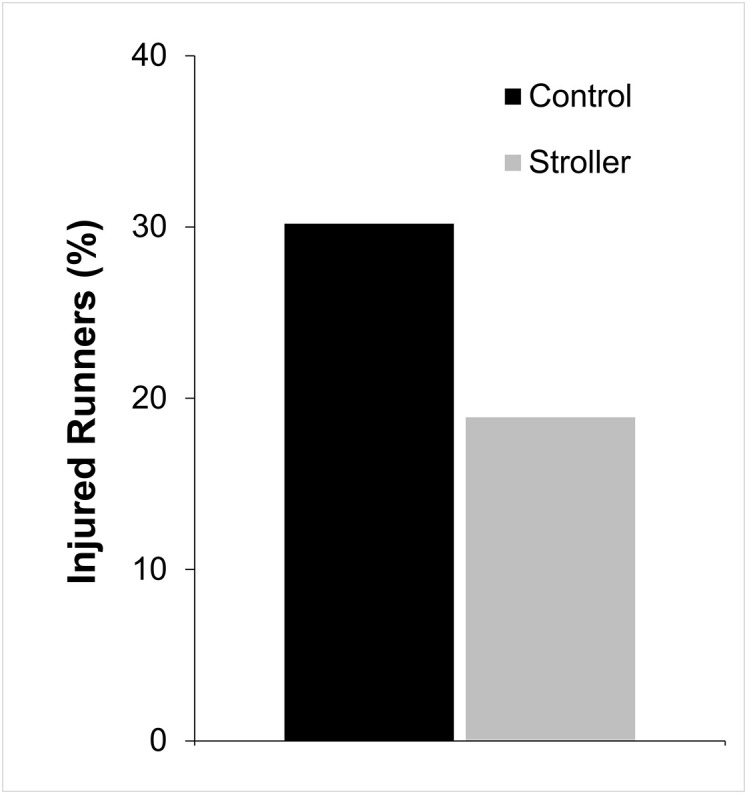
Percentage of injured runners by group. Proportion of running-related injuries sustained by stroller (n = 196) and control groups (n = 53).

**Fig 4:**
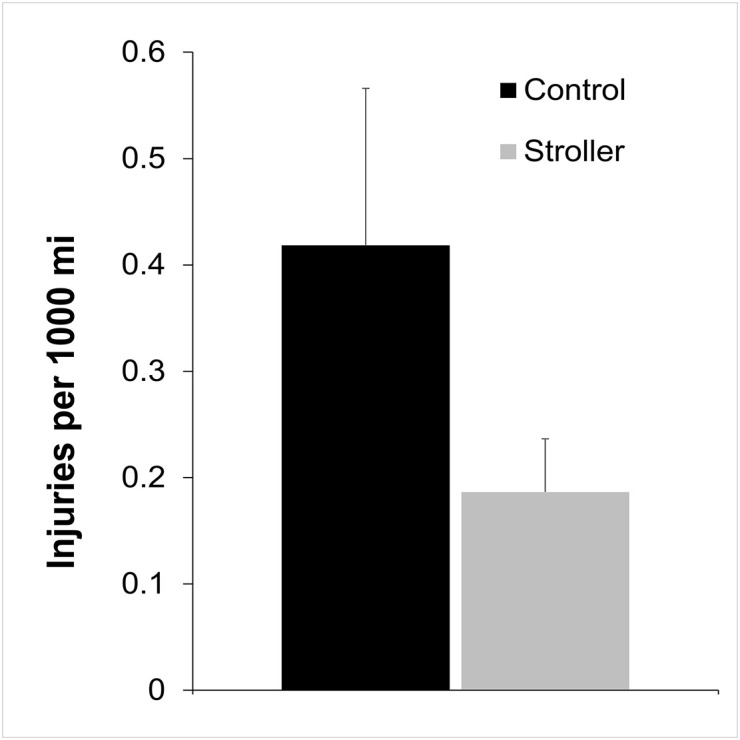
Injury rate per 1000 miles by group. Injuries per 1000 miles in stroller and control groups (stroller = 0.19 ± 0.70, control = 0.42 ± 1.09) when the child is 0-3 years old. Error bars indicate SE.

The incidence rate and distribution of overuse injuries differed between the stroller and control groups (Fig 3). The distribution of injured runners in the control was proportionately more than those in the stroller group and was associated with a small effect size (*V =* 0.11); however, this difference did not meet statistical significance based on the Chi-Squared test (*p* = 0.074, ꭓ^2^ = 3.19). Among stroller runners, 159 of the 196 participants (81%) remained injury-free during the study period, while 37 (19%) reported sustaining a running-related injury. In the comparatively smaller control group, 70% of non-stroller runners remained injury-free, while 30% reported experiencing an overuse injury. A *post hoc* power analysis revealed that this comparison reached a power of 0.99, indicating sufficient power. Among individuals who incurred injuries when their child was 0–3 years old, stroller runners experienced a 55% lower injury rate of 0.19 per 1,000 miles run (95% CI [0.09, 0.28]), compared to 0.42 injuries per 1,000 miles in the control (95% CI [0.13, 0.55]) (Fig 4) (*p* = 0.05, U = 4873.5). While this did not meet a *p* < 0.05 criteria, the difference was associated with a small effect size (*d* = 0.29). A *post hoc* power analysis indicated that this comparison failed to reach the *a priori* alpha level of 0.05 and instead yielded a power of 0.44, suggesting an insufficient sample size to identify a significant difference.

**Fig 5:**
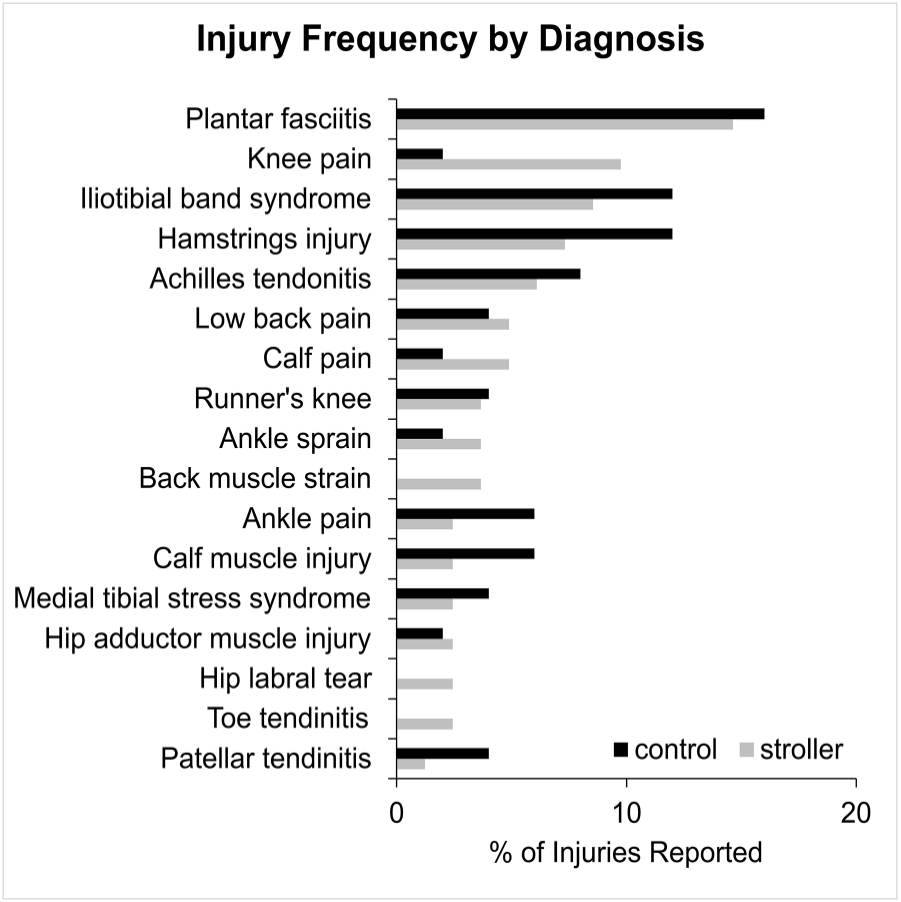
Distribution of reported injuries among stroller and non-stroller runners. 2 or more injuries were reported for each type. Each bar represents the percentage of all injuries reported in the control or stroller group.

The breakdown of injury types were similar in both groups, with the exception of a large incidence of plantar fasciitis, accounting for 15% (n = 12) of all injuries in the stroller group and 16% (n = 8) of all injuries in the control group (Fig 5). In addition to the injuries shown in Fig 5, the incidence of the following injuries were reported once in the stroller group: tibial stress fracture, costal fracture, meniscus injury, metatarsalgia, patellofemoral syndrome, peroneal tendinitis, extensor hallucis longus tendinitis, hip trochanteritis, shin splints, popliteus tendinosis, femur stress fracture, broken foot bone, SI joint dysfunction, and ankle dorsiflexor. In the control group, beyond the injuries reported in Fig 5, the following injuries were each reported once: metatarsalgia, hip trochanteritis, hip adductor tendonitis, quadriceps muscle pain, ankle inversion injury, gastrocnemius muscle injury, tibialis posterior tendinitis, and metatarsal stress fracture.

**Fig 6:**
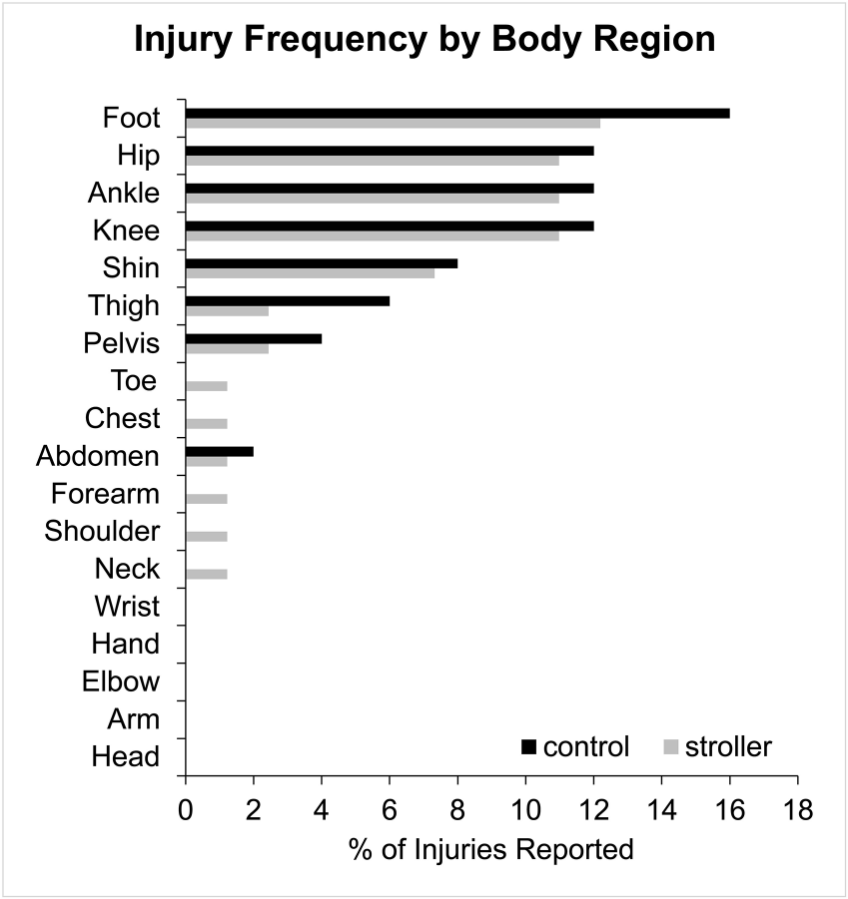
Frequency of overuse injuries by anatomical region reported. Each bar represents the percentage of all injuries reported in the control or stroller group.

Likewise, the regions of the body affected by injury were consistent across groups when considering the total number of participants in each group (Fig 6). The foot was the most frequently injured body part in both groups (stroller: 18%; control: 22%), followed by the hip, ankle, and knee, each accounting for 17% of injuries. Shin injuries were also similar between groups, whereas thigh and pelvic injuries were more common in the control group. Injuries in the stroller group were more spread out across different body regions, including the toe, chest, forearm, shoulder, and neck.

The frequency of overuse injuries varied between stroller and non-stroller runners. In the stroller group, 18 injured participants reported experiencing one injury; ten reported two injuries; four reported three injuries; two reported six injuries; and one individual each reported four, seven, or eleven injuries. Within the smaller control group, two injured runners indicated sustaining a single injury; seven indicated two injuries; five indicated three injuries; and one participant each indicated four, six, or eight injuries. The control group overall experienced a higher incidence of multiple injuries than the stroller group. Four or more injuries were reported by 18% of control runners, compared with 14% of stroller runners. 29% of the control group reported three injuries, while only 11% of the stroller group did. Two injuries were reported by 41% of control runners, relative to 27% of stroller runners.

## Discussion

This study aimed to evaluate how stroller running influences injury risk among parent runners. Our hypothesis that stroller runners may sustain fewer running-related injuries compared to non-stroller runners, owing to varied running patterns and reduced impact load was supported. Specifically among the participants, a lower proportion of stroller runners reported being injured than in the control group, and this difference persisted when considering mileage. The 55% reduction in injury rates per 1000 miles suggests that pushing a stroller may reflect a lower observed injury risk among parent runners by encouraging pace regulation and consistent cadence – both of which have been shown to reduce stress on weight-bearing joints [35]. In addition, previous work suggests that applying downward pressure on the handlebars helps transfer some of the impact forces to the stroller wheels, minimizing the load absorbed by the legs [15]. This reduced impact may be associated with a lower risk of running injuries, such as shin splints, runner’s knee, and stress fractures [5].

Weekly running mileage differed among parent runners according to the child’s age. When children were between 6–12 months old, stroller runners ran more miles than the control group, perhaps because babies can often be securely placed in jogging strollers around six months, enabling parents to incorporate exercise while including their child. This age range may be especially conducive to stroller running, as infants are generally less active and more amenable to time in the stroller, unlike older children. Conversely, between 0–6 months, stroller runners ran less miles with a stroller than without one, as most jogging strollers are not recommended for use until infants can independently support their head and neck. Pediatric safety guidelines often caution against stroller running during this developmental period, limiting parents from running with a stroller until their child is at least 6 months old.

Moreover, stroller running peaked when children were between 12–36 months – presumably because toddlers at this stage have improved trunk and neck control, making stroller use safer and more suitable for accompanying parents during runs. This may also reflect parents becoming more accustomed to their fitness regimen, as children are settling into regular routines and temperaments during this phase of life. However, during this period, stroller runners did run more miles without a stroller than with one, possibly due to toddlers being less tolerant to prolonged stroller confinement, which may constrain the duration of stroller running or discourage stroller use altogether. This phase often coincides with parents resuming their regular exercise routines, supporting the observed increase in mileage during this period. Additionally, by the time their children reached ages 3–10, a majority of stroller runners ran more miles without a stroller – likely due to the growing independence and size of children at this stage, enabling parents to resume conventional running without the need for a stroller.

Among injured participants, both stroller runners and non-stroller runners most frequently reported injuries to the feet – particularly plantar fasciitis. The different distribution of injury diagnoses across groups may be partly attributed to modified running mechanics associated with stroller use, such as increased trunk lean, reduced trunk rotation, and greater anterior pelvic tilt and hip flexion [15]. Such changes may redistribute mechanical stress along the kinetic chain and influence ground reaction force [15]. Likewise, the increased physiological indices of pushing a stroller (e.g., accelerated heart rate and perceived exertion) might be associated with greater running intensity and could potentially predispose runners to running-related injuries like plantar fasciitis [21,36]. In addition, existing evidence shows that runners with greater body mass and lower foot muscle strength experience increased dynamic plantar pressure, which may help explain elevated stress on the plantar fascia and subsequent foot injury risk [37].

Given that both groups exhibited a high incidence of plantar fasciitis, this may be more attributable to postpartum physiology and exercise routines. Some evidence suggests that the incidence of plantar fasciitis increases during pregnancy due to hormonal fluctuations affecting the elasticity of connective tissues and weight gain [37,38]. It is possible that these morphological changes persist postpartum, increasing the incidence of plantar fasciitis in this sub-population. Irregular or inconsistent running patterns can increase the risk of injury and tissue damage [22,23], which may be true of both groups while parenting children under the age of three. Aside from the elevated incidence of plantar fasciitis, the rate and distribution of injuries in both groups were consistent with previous literature, which has established that the bulk of running injuries occur in the lower extremities [9,15].

Importantly, 90% of stroller runners were primarily female, while 73% of non-stroller runners identified as a woman. The role of sex on overuse injuries remains a topic of debate, with some studies suggesting a higher incidence of knee injuries in females [39,40]. All female runners also identified as the birthing parent, and associated postpartum hormonal changes – most notably increased estrogen and progesterone levels – may have reduced connective tissue pliability, thereby heightening their injury risk profile [29]. In addition, female parents may be more prone to engage in stroller running, as women more often assume primary caregiving roles compared to men [41]. Thus, women might be more inclined to spend more time with their children while running. Moreover, the survey was largely promoted through social media platforms with predominantly female users, which may have exacerbated the sex imbalance observed during recruitment for this study. While this study did not query socioeconomic status or cultural identity, it is possible that the demographic profile of our sample was skewed toward individuals with higher socioeconomic status. This may limit the generalizability to parents from different socioeconomic and cultural backgrounds, and future research should seek to include a more diverse population.

Several limitations should be noted. More than half of the initial participants were excluded from the final data analyses, resulting in 303 participants being removed from the study due to not meeting our inclusion criteria or providing incomplete responses. This may have led to a selection bias, whereby the responses reflected a population with a greater interest in reporting and evaluating their running. Additionally, the underrepresentation of injured runners who discontinued running may have resulted in survivorship bias. The relatively smaller sample size – specifically within the control group – may also impose constraints on the statistical significance of the findings, making it difficult to draw definitive conclusions regarding differences between the two groups. However, it is worth mentioning that 79% of respondents chose to run with a stroller at least some of the time. This implies that stroller running may be popular and accessible for parents of young children.

Moreover, the gender imbalance, with 90% of stroller runners being female, may limit the generalizability of the findings to male parent runners given the gender composition of the sample. In addition, the dependence on retrospective self-reported data over a postpartum period of up to 20 years may have introduced recall errors in reporting running mileage and injury details. The survey was designed to avoid asking specific questions regarding injury diagnosis to minimize the effects of significant recall bias, however, this limited the ability to perform multivariate analysis with potential confounding factors such as training history and age. While this survey design was based on previous work [33,34], it was not formally validated for this sub-population which could have increased the risk of self-report bias among survey respondents. The cross-sectional design of the study also restricts the confidence in ascertaining causal relationships between stroller use and injury risk, as temporal associations and changes in training patterns relative to injury onset were not recorded. Furthermore, the study did not measure or control for several key potential confounders, including training intensity, footwear, running surface, and cross-training habits, which may limit interpretation of the observed differences between groups.

Nevertheless, this study offers valuable preliminary insights into the experiences of parent runners and underscores how stroller running may support continued physical activity during early parenthood. In light of the exploratory nature of this study, the findings should be interpreted with caution. Future work should include more demographically balanced cohorts, employ objective methods for assessing running habits and injury occurrence, and conduct detailed analyses of stroller pushing mechanics to better identify and understand potential risk factors.

This is among the first studies to systematically investigate injury patterns and running behaviors among parent runners. These findings suggest that stroller running does not increase the risk of overuse injuries and may be a relatively safe form of exercise for most parents. Additionally, it may serve as a feasible way to maintain fitness during the early years of their children’s lives. A deeper understanding of injury risks and kinematic adaptations within this sub-population may provide a foundation for advancements in evidence-based recommendations for postpartum and parent runners, influence the development of ergonomically optimized running strollers, and contribute to the establishment of tailored injury prevention programs. Given that multi-modal therapeutic interventions can effectively reduce running-related injuries among recreational runners [8,42], adapting similar accessible prevention strategies for parent runners – modified for stroller running biomechanics and postpartum physiology – could minimize overuse injury risk in this sub-population. Ultimately, such efforts may help parents to remain physically active amidst the responsibilities of caring for young children. Further validation in prospective cohorts using objectively measured running mileage and injury surveillance is warranted.
