# Supplementary material for: Injury risk and running habits in parents: Does stroller use make a difference?
Source: PLoS One. 2026 Aug 12;21(8):e0354371. doi: 10.1371/journal.pone.0354371 (PMC13465853; doi:10.1371/journal.pone.0354371)
Supplement: Supplementary file 1 [file pone.0354371.s001.pdf]

Manuscript Number: PONE-D-26-09901 **Title:** Injury risk and running habits in parents: Does stroller use make a difference?

Thank you for the opportunity to review this interesting and timely manuscript addressing an underexplored topic in sports medicine and postpartum physical activity. The study addresses a clinically relevant question with generally clear writing. However, several methodological, statistical, and reporting issues require revision to meet PLOS ONE's standards for observational research. Below are my detailed comments organized by manuscript section.

- **Line 23-41** : The abstract does not explicitly state that this is a cross-sectional study. Please add "cross-sectional survey" or "observational cross-sectional study" to the abstract to help readers immediately understand the study design and its inherent limitations regarding causal inference.
- **Lines 34-35**: Injury rates are reported (0.19 vs. 0.42 per 1,000 miles) but without 95% confidence intervals.
- **Lines 31-32**: Final sample sizes per group are not reported in the abstract. Please include (1) 95% CIs for injury rates, and (2) final analytic sample sizes (Stroller: n=196; Control: n=53) in the abstract to improve transparency and allow readers to assess precision.
- **Lines 62-73**: The literature review discusses biomechanical risk factors but does not explicitly address sex-specific injury patterns, despite 90% of the stroller group being female (Table 1, Line 207). Please expand the background to briefly discuss known sex differences in running injury epidemiology (e.g., higher patellofemoral pain prevalence in women) to better contextualize your predominantly female sample.
- **Lines 135-141**: The hypothesis states stroller runners may have fewer injuries "due to running mechanics and reduced impact load," but the study does not measure biomechanical variables. Please reframe the hypothesis to reflect that it is informed by prior biomechanical literature rather than directly tested in this study, to avoid implying mechanistic causality that the survey design cannot support.
- **Lines 171-173**: The between-subjects design is mentioned, but the cross-sectional nature is not explicitly stated until the Discussion (Lines 392-394). Please state "cross-sectional design" in the Methods section (e.g., Line 171) to align with STROBE and help readers interpret findings appropriately from the outset.

- **Lines 144-145**: Recruitment period is stated (May 2024–Nov 2025), but geographic scope is vague ("email invitations to local running clubs and social media"). Please specify the geographic region(s) targeted for recruitment (e.g., U.S.-based, Pennsylvania-focused, or international) to clarify the study's contextual boundaries and generalizability.
- **Lines 194-201**: Participant flow is described, but it is unclear which group(s) the 6 participants excluded for "insufficient data" belonged to. Please clarify in the Results or a CONSORT-style flow diagram whether the 6 exclusions were from the stroller group, control group, or both, to improve transparency in sample derivation.
- **Lines 168-169**: "Overuse injury" is not operationally defined (e.g., self-reported pain vs. clinician diagnosis, minimum duration, impact on training).
- **Lines 167-169**: Potential confounders (training intensity, footwear, running surface, cross-training habits, prior injury history) are mentioned in the Introduction but not measured or adjusted for in analyses. Please (1) provide a clear operational definition of "overuse injury" used in the survey, and (2) acknowledge in Limitations that key confounders were not measured or controlled, which may affect interpretation of group differences.
- **Lines 144-170**: The survey is described as researcher-developed, but no information on content validity, pilot testing, or test-retest reliability is provided. Please add a brief statement regarding instrument development (e.g., expert review, cognitive testing, or reference to validated items) to support the credibility of self-reported measures.
- **Lines 168-169, 391-392**: The study acknowledges recall bias in the Discussion but does not describe any methodological steps taken to minimize it (e.g., limiting recall period, using memory aids). Please describe any strategies used to reduce recall bias (if any), or explicitly acknowledge in Limitations that retrospective self-report over up to 20 years postpartum may introduce substantial recall error.
- **Lines 194-201**: Sample size resulted from convenience sampling via social media; no a priori power calculation is reported. Please acknowledge that the study used a convenience sample without formal power calculation, and consider reporting a post-hoc power analysis for the primary injury rate comparison to help readers interpret null or trend-level findings.
- **Lines 174-181**: Continuous variables are reported as mean ± SD, but normality testing (Shapiro-Wilk) led to use of non-parametric tests. Please clarify which specific non-parametric test (Mann-Whitney U or Wilcoxon) was applied to each

outcome variable, and report corresponding test statistics (e.g., U value, Z value) alongside p-values for reproducibility.

- **Lines 179-191**: Statistical methods are described, but:
    - No multivariable analysis is performed to adjust for potential confounders (sex, age, prior injury, training habits).
    - Multiple subgroup comparisons (injury types, anatomical regions) are conducted without correction for multiple testing. Please (1) consider adding a multivariable logistic regression model to estimate adjusted odds of injury, and (2) apply a correction for multiple comparisons (e.g., Bonferroni, FDR) or explicitly acknowledge the increased risk of Type I error in subgroup analyses.

Lines 194-201, Fig 1: Participant flow is generally clear, but:

- The 6 exclusions for "insufficient data" (Line 200) are not allocated to specific groups.
- The reason for excluding 36 participants who "did not resume running" (Line 197) could be clarified (were they ineligible or simply not relevant to the research question?). Please revise Fig 1 or the accompanying text to specify group allocation for all exclusions and clarify whether non-runners were excluded a priori or post-hoc.

- **Lines 200-201**: No information is provided on the extent or pattern of missing data for key variables (e.g., injury history, mileage). Please report the proportion of missing data for primary outcomes and, if >5%, describe any sensitivity analyses or assumptions made about missingness (e.g., missing completely at random).
- **Lines 263-264, 272-273**: Injury rates are reported, but:
    - There is an inconsistency: the text reports p=0.05 (Line 264), while Figure 4 caption describes this as "a trend towards significance (p<0.1)" (Line 273).
    - Error bars are reported as SD in Figure 4 but as SE in Figure 2 (Line 240), without justification. Please (1) reconcile the p-value reporting discrepancy between text and figure captions, and (2) standardize error bar metrics across all figures (preferably 95% CI for inferential clarity) or provide methodological rationale for using different metrics.
- **Lines 257-264**: Unadjusted comparisons are reported, but no adjusted analyses are presented despite potential confounding by sex, age, or training history. Please

present at least one multivariable model adjusting for key demographic and training-related confounders to help readers assess whether observed group differences persist after accounting for these factors.

- **Lines 243-255**: Mileage patterns across child age groups are descriptively presented, but no formal interaction tests (e.g., group × child age) are reported. If exploratory subgroup analyses were conducted, please label them as such and consider reporting interaction p-values to formally test whether stroller use effects differ by child age or other modifiers.
- **Lines 318-328**: Key findings are summarized, but causal language ("confer a safeguarding effect," Line 323; "transfer impact forces," Line 327) exceeds what a cross-sectional design can support. Please revise causal interpretations to associative language (e.g., "was associated with lower injury incidence") to align with the observational design and limitations acknowledged in Lines 392-394.
- **Lines 386-394**: Several limitations are acknowledged (sample size, self-report, cross-sectional design), but:
    - Survivorship bias (injured runners who quit running may be underrepresented) is not mentioned.
    - The potential impact of gender imbalance (90% female in stroller group) on generalizability to male parents is not discussed. Please expand the Limitations section to explicitly address (1) potential survivorship bias, and (2) limited generalizability to male parent runners given the gender composition of the sample.
- **Lines 396-409**: Conclusions emphasize clinical utility but could better temper claims given methodological constraints. Please strengthen the interpretation by explicitly linking conclusions to the study's exploratory nature and suggesting that findings require validation in prospective, objectively measured cohorts.
- **Lines 368-378, 396-409**: The sample is described as predominantly White, educated, and female, but no explicit discussion of how this limits applicability to more diverse populations is provided. Please add a brief paragraph discussing how the demographic profile of your sample may limit generalizability to parents from different cultural, socioeconomic, or geographic backgrounds.
- **Lines 146-153**: IRB exemption is noted, but it is unclear whether participants were informed of this status during consent. Please clarify in the Methods whether the

informed consent statement explicitly mentioned the IRB exemption determination, to ensure transparency in the ethical approval process.

Figure Reporting Consistency

- **Figure 2 caption (Line 240)**: Error bars = SE
- **Figure 4 caption (Line 273)**: Error bars = SD
- Please standardize error bar reporting across all figures (SE or 95% CI preferred for group comparisons) to avoid visual misinterpretation and align with STROBE/PLOS ONE reporting standards.
- **Line 323**: "This 79% difference suggests..." lacks clear derivation. Injury prevalence was 19% vs. 30% (absolute difference 11 percentage points; relative difference ~37%). Please verify the calculation underlying the "79% difference" claim or revise this interpretation to accurately reflect the observed effect size (e.g., "37% lower relative injury prevalence").

- It is recommended that the authors enrich the Introduction and Discussion sections by incorporating recent evidence on multifactorial determinants of running-related injuries and evidence-based prevention strategies. Specifically, consider citing:

Bagheri, Shahabeddin, et al. "Adding mindfulness practice to exercise therapy for female recreational runners with patellofemoral pain: a randomized controlled trial." *Journal of athletic training* 56.8 (2021): 902-911.

Naderi, Aynollah, et al. "Obesity and foot muscle strength are associated with high dynamic plantar pressure during running." *The Foot* 44 (2020): 101683.

Naderi, Aynollah, Maarten H. Moen, and Hans Degens. "Is high soleus muscle activity during the stance phase of the running cycle a potential risk factor for the development of medial tibial stress syndrome? A prospective study." *Journal of sports sciences* 38.20 (2020): 2350-2358.

Naderi, Aynollah, et al. "Predictors of running-related injury among recreational runners: a prospective cohort study of the role of perfectionism, mental toughness, and passion in running." *Sports health* 16.6 (2024): 1038-1049.

Naderi, Aynollah, et al. "Effects of integrating lower-leg exercises into a multimodal therapeutic approach on medial tibial stress syndrome management among recreational

runners: a randomized controlled study." *Orthopaedic journal of sports medicine* 13.2 (2025): 23259671241311849.

Naderi, Aynollah, Ali Yasemi, and Seyed Hamed Mousavi. "Effectiveness of an online multi-component exercise-based injury prevention program on running-related injury risk in recreational runners: a randomized controlled trial." *Research in Sports Medicine* 34.2 (2026): 217-237.

Integrating these references would enhance the theoretical depth, clinical relevance, and global applicability of your manuscript.
