# Supplementary material for: Injury risk and running habits in parents: Does stroller use make a difference?
Source: PLoS One. 2026 Aug 12;21(8):e0354371. doi: 10.1371/journal.pone.0354371 (PMC13465853; doi:10.1371/journal.pone.0354371)
Supplement: Supplementary file 2 [file pone.0354371.s003.docx]

Thank you for your thorough review of this manuscript. Our responses to the editor and reviewers’ comments are shown in red text. We appreciate the time and effort that went into each of these comments and have made all of the requested changes possible given the data set collected.

Journal Requirements:

When submitting your revision, we need you to address these additional requirements.

1. Please ensure that your manuscript meets PLOS ONE's style requirements, including those for file naming. The PLOS ONE style templates can be found at

<https://journals.plos.org/plosone/s/file?id=wjVg/PLOSOne_formatting_sample_main_body.pdf> and

<https://journals.plos.org/plosone/s/file?id=ba62/PLOSOne_formatting_sample_title_authors_affiliations.pdf>

We have adapted the manuscript and figure naming format to meet the journal’s formatting requirements.

2. When completing the data availability statement of the submission form, you indicated that you will make your data available on acceptance. We strongly recommend all authors decide on a data sharing plan before acceptance, as the process can be lengthy and hold up publication timelines. Please note that, though access restrictions are acceptable now, your entire data will need to be made freely accessible if your manuscript is accepted for publication. This policy applies to all data except where public deposition would breach compliance with the protocol approved by your research ethics board. If you are unable to adhere to our open data policy, please kindly revise your statement to explain your reasoning and we will seek the editor's input on an exemption. Please be assured that, once you have provided your new statement, the assessment of your exemption will not hold up the peer review process.

The data are now available at <https://osf.io/et7nz/>.

3. If the reviewer comments include a recommendation to cite specific previously published works, please review and evaluate these publications to determine whether they are relevant and should be cited. There is no requirement to cite these works unless the editor has indicated otherwise.

Additional citations have been added to reflect the editor and reviewers’ suggestions.

Additional Editor Comments:

A major concern is that the conclusions are not fully supported by the data. The authors need to revise the manuscript to better align conclusions with data:

1. Figs 3 +4: The authors describe the data as demonstrating "a trend toward significance." While this is techinically correct, I would prefer the authors not support this data with a non-significant p-value (p<0.1). A better place to discuss the "trend toward significance" and perhaps discuss the reason for this (likely sample size was underpowered to see a difference) would be in the discussion.

Thank you for this comment. We have removed the # indicators from the graphs and captions, and stated in the text that these results did not meet the criteria for statistical significance. We have included effect size estimates instead to demonstrate the meaning of the data presented.

2. The conclusion "stroller running is generally safe and accessible form of exercise for parent runners, with lower injury incidence observed compared to non-stroller runners," needs to be revised in light of point 1.

We see your point here; however, even the lack of statistically significant findings supports that there is no increase in running injury risk when running with a stroller. We’ve added text to clarify that the injury rates between groups were not different, indicating that running with a stroller is safe in the text to ensure the full message is communicated.

3. As a reviewer pointed out- the authors need to revise the discussion around incidence of plantar fasciitis. The text reads as though there were significant differences in this injury across groups, which is not supported by the data- I think the point is that the overall cohort had a notable high incidence of plantar fasciitis- is there evidence for higher risk of this injury among pregnant/early postpartum women?

There is some evidence that suggests incidence of plantar fasciitis may be affected by pregnancy (Foraldy et al., 2024). Whether that is due to hormonal fluctuations or weight gain is unclear, however, some evidence exists to suggest that plantar fascia thickness increases (Foraldy et al., 2024) and ligaments experience more laxity (Chu et al., 2019) during pregnancy. These morphological changes persist even after pregnancy, and thus, may in part cause this increase in plantar fasciitis observed in this population. References and text have been added to the manuscript to reflect this and clarify the concept.

5. Review Comments to the Author

Please use the space provided to explain your answers to the questions above. You may also include additional comments for the author, including concerns about dual publication, research ethics, or publication ethics. (Please upload your review as an attachment if it exceeds 20,000 characters)

Reviewer #1: Thank you for inviting me to review this interesting paper. The topic is very novel and important and is worthwhile research. I would like to commend the authors for researching in this important area. I believe the authors could make the abstract clearer to readers that this was solely a cross-sectional survey and not a longitudinal study as is somewhat implied in my opinion. The methods require improved clarity, the specific variables queried to runners is unclear and impacts the understanding of the results section. Also was a sample size calculation completed?

We thank the reviewers for this thorough review of the manuscript. The changes mentioned have been addressed here and in the comments below individually, with associated changes in the manuscript. We believe these changes to clarify the study design have helped to strengthen the manuscript.

Please see information below that I hope the authors find useful.

Abstract:
1. Lines 36-38: Injury type and distribution were similar between groups, although there was a higher incidence of plantar fasciitis reported among both stroller and non-stroller runners.” – not sure why this is led in as an although, if it was high in both it should be “and” instead perhaps?

We agreed and separated these two sentences to improve clarity and removed the “although” to prevent confusion.

2. From reading later in the paper, this is a cross-sectional survey. This is not clear in this abstract and it implies that it is a 1.5 year longitudinal study.

That is true. We have added that the study was a cross-sectional design in the abstract and clarified the language to ensure it is clear that subjects were recruited over a 1.5 year period, and not tracked over this period.

3. The number of participants should be reported in the abstract.

The group participant numbers have been added in the abstract.

4. The comparison between injury rate per 1,000 miles should clarify that it includes those with children aged 0-3 years as that is not clear

Text was added to the abstract to clarify that injuries were assessed from the first three years of parenting.

Introduction:
1. Lines 56-60: This sentence is quite hard to read and I am not sure what it adds really beyond the specific injuries noted earlier in the introduction section. I would consider removing this. I think solely from “Since about….” Is sufficient here.

The sentence in question was deleted and edits were made to the surrounding sentence to adjust the flow and clarify the injury breakdown.

2. Lines 129-131: “shown that decrease in..” – add an “a” there a decrease in.

Good catch, we added the “a” in this sentence.

Methods
1. It is slightly confusing to me that the start of the paragraph 1 in the methods section says it is exempt from ethics review but then later discusses what was approved?

Our apologies for the lack of clarity here; this was text we were required to add due to journal guidelines, and it doesn’t really make sense with an exempt study. I added that the consent method was approved “as exempt” from the institutional review board in the final sentence to ensure the statements are not contradictory.

2. The abstract implies that they were tracked for 1.5 years – whereas in reality this is a once off cross-sectional survey right? This is very unclear to the reader.

Clarifying text was added to the first paragraph of the methods section and the abstract to indicate that this was a cross-sectional study.

3. I am also very unclear about the period they are being asked about in the study. So to be eligible they had to have been a parent to children aged between 0-3 years within the last 20 years. So am I correct that this study is assessing people’s injuries just over a certain amount of time during that period? Or since then? The methods section is really lacking clarity on for what period the participants are reporting related to their injuries etc.

A sentence was added to clarify that runners were asked about their injuries during the period of time that is most commonly associated with stroller running, when their children were between 0 and 3 years of age.

4. Was a sample size calculation completed for this study? Or a power calculation completed after the study was completed?

An *a priori* power analysis was not completed at the onset of the study due to a lack of understanding of what we might find. *Post hoc* power analysis revealed that we were sufficiently powered in our Chi Squared calculation of the proportion of injured vs. healthy runners in each group, but may not have been sufficiently powered to observe a difference in injuries per 1000 miles run. We have made modifications to the text to reflect this.

5. I am unable to comment on the statistical analysis utilised as I am not clear what data was exactly collected from the methods section. So while in the form I ticked no, I am not saying the data is incorrect, I just was unable this aspect based on the information above.

Text was added to clarify the statistical methods used, and the data were provided via Open Science Framework <https://osf.io/et7nz/>.

Results
1. Did you consider the impact of gender in the different groups?

We considered the potential impact of gender in our analyses; however, both groups were predominantly composed of female runners, and the relatively small number of male participants limited meaningful gender-based comparisons. We assessed that the higher participation of female runners may itself be a notable finding, potentially reflecting that mothers within the parent runner population are more likely to identify as parents who run and participate in stroller running with their children, as they are more commonly the primary caregivers.

2. Lines 258-260: I don’t think there is a need to consider “trending towards significance” – it was 0.07 and so was non-significant and should be stated as such.

You are right in pointing this out. We have removed the phrase “trending towards significance” and revised the statement to appropriately report this result as not statistically significant. We have also added the calculated effect size to demonstrate a medium effect.

3. Lines 276-279: This can be streamlined into one sentence.

We have streamlined the text into a single sentence, improving conciseness.

4. Lines 276-290: This is a very long detailed paragraph that doesn’t really add much beyond what is outlined in a figure. This needs to be condensed.

We have condensed this paragraph to eliminate redundancy, and eliminated redundant information that can be viewed in Figure 5.

5. What were the 95% CI for both groups for injury rates per 1,000 miles? That should be reported. Also solely 0-3 years was compared, is this because only injury during this time period was asked was it? The lack of clarity in the methods is impacting understanding of the results in my opinion and this should be rectified.

The 95% confidence intervals for the stroller group were [0.09, 0.28] and the control group [0.13, 0.55]. This data has been added to the results for clarity. Additional text was added to the methods regarding the period of time requested.

Discussion
1. Lines 269-370. Did you query sex or gender in the survey? The terms are being used interchangeably here and should align with what was specifically asked in the survey.

Sex was queried from participants, and this term was used throughout the manuscript. “Gender bias” was used to describe the imbalance in recruitment of males vs. females, and this sentence has been corrected for clarity to avoid confusion between sex and gender.

2. Lines 380-386. This is a typical issue with surveys. It should be part of the limitations section and shortened to one/two lines and condensed.

We have moved the text to the limitations section and condensed it to two lines.

Note: There is a query on the review submission regarding the data being fully available. The statement in this document says that it will be published once accepted but I do not have access to it currently.

The data has been made available through Open Science Framework for the next round of review. It can be found at <https://osf.io/et7nz/>.

Reviewer #2: Thank you for submitting this timely and clinically relevant manuscript examining injury risk among parent runners who use strollers—a notably underexplored population in sports medicine. The study addresses an important public health question with generally clear writing and appropriate descriptive analyses. However, several methodological and reporting issues require revision before publication.

Thank you for your review of our work. We have attempted to address the methodological and reporting issues below. For clarity in our response, we separated out the reviewer’s comments into each topic and responded underneath.

The cross-sectional, self-reported survey design inherently limits causal inference, yet the discussion employs causal language (e.g., "confer a safeguarding effect") that overstates what the data can support; please revise to associative phrasing throughout.

We have revised the manuscript throughout, especially the discussion section, to remove causal language and replaced it with appropriate associative phrasing to better reflect the limitations of our study design and avoid overstatement of the data provided.

Key statistical reporting inconsistencies should be resolved, including the discrepancy between p=0.05 in the text versus "trend (p<0.1)" in Figure 4

The trend toward significance text has been removed throughout the manuscript based on the reviewers’ suggestions. The p-value of 0.05 is still included in the text along with an effect size calculation, demonstrating a small effect size.

inconsistent error bar metrics (SE vs. SD),

We’ve made changes for consistency, making all variation representations standard error rather than standard deviation (Table 1, Figure 2, and Figure 4).

and the unexplained "79% difference" claim.

The authors thank the reviewers for catching this. We retraced our steps and clarified the percentage of interest in the results first, then repeated the reduction in injury rate in the discussion.

The operational definition of "overuse injury" requires clarification

An operational definition of overuse injury has been added to the methods section indicating that any self-reported injury was considered for this study without consideration to clinical diagnosis, pain, duration, or impact on training. This is also discussed as a limitation in the discussion.

the absence of effect sizes, confidence intervals

Effect sizes and confidence intervals have been added throughout the text wherever appropriate.

multivariable adjustment for potential confounders (training intensity, prior injury, footwear) limits interpretability.

We have clarified in the Limitations section in our discussion that unmeasured confounding variables may have influenced the observed group differences.

Additionally, please explicitly state the cross-sectional design in the abstract and methods per STROBE guidelines

We have revised the abstract and methods to explicitly state the cross-sectional design of our study per STROBE guidelines.

address the gender imbalance (90% female in stroller group) and its implications for generalizability

We have included a limitation in the discussion addressing the gender imbalance and noting that generalizability to male parent runners may be limited given the predominantly female composition of the sample.

clarify instrument validation for the researcher-developed survey.

The study is based on surveys used in previous studies by Altman & Davis 2012, and Pohl et al., 2008. These references and descriptions have been added to the manuscript for clarity. It was also added to the discussion that this specific survey tool was not validated for this particular population to increase transparency.

With these revisions to strengthen methodological transparency, statistical rigor, and interpretive caution, this manuscript has strong potential to make a valuable contribution to the literature on postpartum physical activity and injury prevention. Recommendation: Major Revision.

6. PLOS authors have the option to publish the peer review history of their article ([what does this mean?](https://track.editorialmanager.com/CL0/https:%2F%2Fjournals.plos.org%2Fplosone%2Fs%2Feditorial-and-peer-review-process%23loc-peer-review-history/1/010f019d941bb4a9-8b83eda8-30fe-4327-9683-0638452d00fd-000000/YncL2FydlP3w3N6rYCzF5V2GBD-R6jykpaxoq9DfsF8=258)). If published, this will include your full peer review and any attached files.


If you choose “no”, your identity will remain anonymous but your review may still be made public.


**Do you want your identity to be public for this peer review?** For information about this choice, including consent withdrawal, please see our [Privacy Policy](https://track.editorialmanager.com/CL0/https:%2F%2Fwww.plos.org%2Fprivacy-policy/1/010f019d941bb4a9-8b83eda8-30fe-4327-9683-0638452d00fd-000000/CHs4NQYQqpFfrTUbWO2GOCD0wtGGHxlpzEosdftp0oE=258).

Reviewer #1: No

Reviewer #2: No

Manuscript Number: PONE-D-26-09901 Title: Injury risk and running habits in parents:

Does stroller use make a difference?

Thank you for the opportunity to review this interesting and timely manuscript

addressing an underexplored topic in sports medicine and postpartum physical

activity. The study addresses a clinically relevant question with generally clear

writing. However, several methodological, statistical, and reporting issues require

revision to meet PLOS ONE's standards for observational research. Below are my

detailed comments organized by manuscript section.

We thank the editor for their thorough review of the manuscript.

• Line 23-41 : The abstract does not explicitly state that this is a cross-sectional

study. Please add "cross-sectional survey" or "observational cross-sectional study"

to the abstract to help readers immediately understand the study design and its

inherent limitations regarding causal inference.

We have revised the abstract to include the term “cross-sectional study” in the text as suggested.

• Lines 34-35: Injury rates are reported (0.19 vs. 0.42 per 1,000 miles) but without

95% confidence intervals.

95% confidence intervals have been added to this data.

• Lines 31-32: Final sample sizes per group are not reported in the abstract. Please

include (1) 95% CIs for injury rates, and (2) final analytic sample sizes (Stroller:

n=196; Control: n=53) in the abstract to improve transparency and allow readers to

assess precision.

We have included in the abstract the final analytic sample sizes and 95% confidence intervals.

• Lines 62-73: The literature review discusses biomechanical risk factors but does not

explicitly address sex-specific injury patterns, despite 90% of the stroller group

being female (Table 1, Line 207). Please expand the background to briefly discuss

known sex differences in running injury epidemiology (e.g., higher patellofemoral

pain prevalence in women) to better contextualize your predominantly female

Sample.

The background has been updated to include a summary of injury patterns by sex in the introduction.

• Lines 135-141: The hypothesis states stroller runners may have fewer injuries "due

to running mechanics and reduced impact load," but the study does not measure

biomechanical variables. Please reframe the hypothesis to reflect that it is

informed by prior biomechanical literature rather than directly tested in this study,

to avoid implying mechanistic causality that the survey design cannot support.

We have revised our hypothesis to reflect that it is based on prior biomechanical literature rather than being directly tested in our study, and removed language implying mechanistic causality.

• Lines 171-173: The between-subjects design is mentioned, but the cross-sectional

nature is not explicitly stated until the Discussion (Lines 392-394). Please state

"cross-sectional design" in the Methods section (e.g., Line 171) to align with STROBE

and help readers interpret findings appropriately from the outset.

We have specified that the study is cross-sectional in the Methods section.

• Lines 144-145: Recruitment period is stated (May 2024–Nov 2025), but geographic

scope is vague ("email invitations to local running clubs and social media"). Please

specify the geographic region(s) targeted for recruitment (e.g., U.S.-based,

Pennsylvania-focused, or international) to clarify the study's contextual boundaries

and generalizability.

We have clarified and explicitly stated the geographic regions targeted for recruitment in the Methods section.

• Lines 194-201: Participant flow is described, but it is unclear which group(s) the 6

participants excluded for "insufficient data" belonged to. Please clarify in the

Results or a CONSORT-style flow diagram whether the 6 exclusions were from the

stroller group, control group, or both, to improve transparency in sample derivation.

We have clarified the Results section to specify how many of the 6 total exclusions were from the stroller (n = 4) and control groups (n = 2).

• Lines 168-169: "Overuse injury" is not operationally defined (e.g., self-reported pain

vs. clinician diagnosis, minimum duration, impact on training).

An operational definition of overuse injury has been added to the Methods section indicating that any self-reported injury was considered for this study without consideration to clinical diagnosis, pain, duration, or impact on training. This is also discussed as a limitation in the Discussion.

• Lines 167-169: Potential confounders (training intensity, footwear, running surface,

cross-training habits, prior injury history) are mentioned in the Introduction but not

measured or adjusted for in analyses. Please (1) provide a clear operational

definition of "overuse injury" used in the survey, and (2) acknowledge in Limitations

that key confounders were not measured or controlled, which may affect

interpretation of group differences.

We have operationally defined “overuse injury” as described above. In addition, we have acknowledged in the Limitations that several key potential confounders were not measured or controlled for, which may limit interpretation of the observed group differences.

• Lines 144-170: The survey is described as researcher-developed, but no information

on content validity, pilot testing, or test-retest reliability is provided. Please add a

brief statement regarding instrument development (e.g., expert review, cognitive

testing, or reference to validated items) to support the credibility of self-reported

Measures.

The survey design was based on work by Altman & Davis (2012) and Pohl et al. (2008) and adapted for this particular population. The manuscript was revised to include this information, and a statement was added to the limitations to indicate that the specific survey instrument was not validated within this population.

• Lines 168-169, 391-392: The study acknowledges recall bias in the Discussion but

does not describe any methodological steps taken to minimize it (e.g., limiting recall

period, using memory aids). Please describe any strategies used to reduce recall

bias (if any), or explicitly acknowledge in Limitations that retrospective self-report

over up to 20 years postpartum may introduce substantial recall error.

We have explicitly acknowledged in the Limitations that the retrospective nature of our self-reported data, encompassing up to 20 years postpartum, may introduce substantial recall errors.

• Lines 194-201: Sample size resulted from convenience sampling via social media;

no a priori power calculation is reported. Please acknowledge that the study used a

convenience sample without formal power calculation, and consider reporting a

post-hoc power analysis for the primary injury rate comparison to help readers

interpret null or trend-level findings.

Text was added to the results section to indicate that the sample collected was a convenience sample. A post hoc power analysis was performed and described in detail with the related statistical reporting provided for both the Chi Squared analysis of the proportion of injured runners and the Mann Whitney U test comparing the rate of injury per 1000 miles.

• Lines 174-181: Continuous variables are reported as mean ± SD, but normality

testing (Shapiro-Wilk) led to use of non-parametric tests. Please clarify which

specific non-parametric test (Mann-Whitney U or Wilcoxon) was applied to each

outcome variable, and report corresponding test statistics (e.g., U value, Z value)

alongside p-values for reproducibility.

For the demographic parameters and mileage, the assumptions for standardized statistical tests were met, so t-tests and chi squared tests were used to determine differences. The assumptions for normality and spread were not met for the calculation of injury rates per 1000 miles. We have clarified in the text that the Mann-Whitney U was used as our non-parametric test for injury rates, and we have reported the corresponding U statistics alongside the p-values for reproducibility.

• Lines 179-191: Statistical methods are described, but:

o No multivariable analysis is performed to adjust for potential confounders

(sex, age, prior injury, training habits).

Text was added to the limitations section that confounding variables may have played a role in injury findings.

o Multiple subgroup comparisons (injury types, anatomical regions) are

conducted without correction for multiple testing. Please (1) consider adding

a multivariable logistic regression model to estimate adjusted odds of injury,

and (2) apply a correction for multiple comparisons (e.g., Bonferroni, FDR) or

explicitly acknowledge the increased risk of Type I error in subgroup

analyses.

Statistical analyses were not performed on the different injury types and anatomical regions due to lack of statistical power, only descriptive comparisons were made, thus no correction was needed.

Lines 194-201, Fig 1: Participant flow is generally clear, but:

o The 6 exclusions for "insufficient data" (Line 200) are not allocated to specific

Groups.

We appreciate you catching this. We have clarified the Results section to specify how many of the 6 total exclusions were from the stroller (n = 4) and control groups (n = 2).

o The reason for excluding 36 participants who "did not resume running" (Line

197) could be clarified (were they ineligible or simply not relevant to the

research question?).

We have clarified the text to specify that the 36 participants were excluded because they could not provide post-parenthood running mileage or injury data relevant to the study’s objectives, which required individuals to have continued running both before and after having a child. These participants were not relevant to the research question described in this paper, but may be used in future research to better understand exercise habits of parents.

Please revise Fig 1 or the accompanying text to specify

group allocation for all exclusions and clarify whether non-runners were

excluded a priori or post-hoc.

The text has been revised to separate the excluded participants into those determined a priori or post hoc. To clarify here, the inclusion criteria for the survey were determined based on a separate study looking at exercise motivation while pushing a stroller, thus the inclusion criteria only establishes that they are a parent of a child and at some point identified as a runner. For this study, we determined post hoc that to be included in the analysis, they must have continued running after becoming a parent and complete a sufficient portion of the survey to determine demographics, mileage, and injury occurrence.

• Lines 200-201: No information is provided on the extent or pattern of missing data

for key variables (e.g., injury history, mileage). Please report the proportion of

missing data for primary outcomes and, if >5%, describe any sensitivity analyses or

assumptions made about missingness (e.g., missing completely at random).

The post hoc inclusion criteria resulted in 255 eligible participants, of which only 6 were removed (2.3%). Thus no additional analyses were required.

• Lines 263-264, 272-273: Injury rates are reported, but:

o There is an inconsistency: the text reports p=0.05 (Line 264), while Figure 4

caption describes this as "a trend towards significance (p<0.1)" (Line 273).

o Error bars are reported as SD in Figure 4 but as SE in Figure 2 (Line 240),

without justification. Please (1) reconcile the p-value reporting discrepancy

between text and figure captions, and (2) standardize error bar metrics

across all figures (preferably 95% CI for inferential clarity) or provide

methodological rationale for using different metrics.

The trend toward significance text has been removed throughout the manuscript based on the reviewers’ suggestions. The p-value of 0.05 is still included in the text along with an effect size calculation, demonstrating a small effect size. We have standardized the error bar metric across all figures to represent standard error.

• Lines 257-264: Unadjusted comparisons are reported, but no adjusted analyses are

presented despite potential confounding by sex, age, or training history. Please

present at least one multivariable model adjusting for key demographic and

training-related confounders to help readers assess whether observed group

differences persist after accounting for these factors.

We appreciate your comment and agree that these are important aspects that affect injury. However, the current data set does not contain sufficient data regarding participant age when the injury occurred and a full training history prior to having children to sufficiently assess this in a multivariate model. In addition, the current data set is heavily skewed toward female participants, so accounting for sex would not allow for additional, meaningful understanding. We have added the lack of multivariate analysis to the limitations section in the discussion.

• Lines 243-255: Mileage patterns across child age groups are descriptively

presented, but no formal interaction tests (e.g., group × child age) are reported. If

exploratory subgroup analyses were conducted, please label them as such and

consider reporting interaction p-values to formally test whether stroller use effects

differ by child age or other modifiers.

Thank you for this suggestion. We overlooked the interaction between group and child age in our initial writeup, but have now included it. Essentially, there was a significant interaction between total mileage run, where stroller runners ran more miles prior to their child reaching 3 years, and this flipped by the time the child reached the 6–10 year age.

• Lines 318-328: Key findings are summarized, but causal language ("confer a

safeguarding effect," Line 323; "transfer impact forces," Line 327) exceeds what a

cross-sectional design can support. Please revise causal interpretations to

associative language (e.g., "was associated with lower injury incidence") to align

with the observational design and limitations acknowledged in Lines 392-394.

We have revised the manuscript throughout, especially the Discussion section, to remove causal language and replace it with appropriate associative phrasing to better reflect the limitations of our study design and avoid overstatement of the data provided.

• Lines 386-394: Several limitations are acknowledged (sample size, self-report,

cross-sectional design), but:

o Survivorship bias (injured runners who quit running may be

underrepresented) is not mentioned.

o The potential impact of gender imbalance (90% female in stroller group) on

generalizability to male parents is not discussed. Please expand the

Limitations section to explicitly address (1) potential survivorship bias, and

(2) limited generalizability to male parent runners given the gender

composition of the sample.

We have expanded the limitations section to explicitly address the potential for survivorship bias and the impact of gender imbalance on the generalizability of the findings to male parent runners.

• Lines 396-409: Conclusions emphasize clinical utility but could better temper

claims given methodological constraints. Please strengthen the interpretation by

explicitly linking conclusions to the study's exploratory nature and suggesting that

findings require validation in prospective, objectively measured cohorts.

Thank you for this thoughtful suggestion. We have revised the Conclusions section to better reflect the exploratory nature of the study and to further temper the interpretation of the findings given the methodological constraints. Specifically, we added language emphasizing that the findings should be interpreted cautiously and clarified that further validation in prospective cohorts using objectively measured running mileage and injury surveillance is warranted.

• Lines 368-378, 396-409: The sample is described as predominantly White,

educated, and female, but no explicit discussion of how this limits applicability to

more diverse populations is provided. Please add a brief paragraph discussing how

the demographic profile of your sample may limit generalizability to parents from

different cultural, socioeconomic, or geographic backgrounds.

This study did not query socioeconomic or cultural backgrounds; therefore, this information is unknown in the present population. However, text was added to the limitations section to acknowledge this gap, as it could limit generalizability.

• Lines 146-153: IRB exemption is noted, but it is unclear whether participants were

informed of this status during consent. Please clarify in the Methods whether the

informed consent statement explicitly mentioned the IRB exemption determination,

to ensure transparency in the ethical approval process.

The consent section of the survey was designed based on HRP-584 Consent Guidance for Exempt Research from the Pennsylvania State University Institutional Review Board. This guidance does not require an explicit statement that the study was exempt, but it does require a minimum of required information, including, but not limited to, the minimal risk, the potential for loss of confidentiality, and the ability to stop at any time. This implied consent method was approved by the PSU IRB with the exempt status designation.

Figure Reporting Consistency

• Figure 2 caption (Line 240): Error bars = SE

• Figure 4 caption (Line 273): Error bars = SD

• Please standardize error bar reporting across all figures (SE or 95% CI preferred for

group comparisons) to avoid visual misinterpretation and align with STROBE/PLOS

ONE reporting standards.

Thank you for bringing this inconsistency to our attention. We have standardized the error bar reporting across all figures to represent standard error in order to reduce potential visual misinterpretation and ensure consistency with STROBE/PLOS ONE reporting standards.

• Line 323: "This 79% difference suggests..." lacks clear derivation. Injury prevalence

was 19% vs. 30% (absolute difference 11 percentage points; relative difference

~37%). Please verify the calculation underlying the "79% difference" claim or revise

this interpretation to accurately reflect the observed effect size (e.g., "37% lower

relative injury prevalence").

The authors thank the reviewers for catching this. We retraced our steps and clarified the percentage of interest in the Results first, then repeated the reduction in injury rate in the Discussion (“55% reduction in injury rates per 1000 miles”).

• It is recommended that the authors enrich the Introduction and Discussion sections

by incorporating recent evidence on multifactorial determinants of running-related

injuries and evidence-based prevention strategies. Specifically, consider citing:

We appreciate your suggestion and have incorporated several additional references, including from those recommended, that align most closely with the content presented in this manuscript. We believe these additions have improved the paper substantially.

Bagheri, Shahabeddin, et al. "Adding mindfulness practice to exercise therapy for female

recreational runners with patellofemoral pain: a randomized controlled trial." Journal of

athletic training 56.8 (2021): 902-911.

Naderi, Aynollah, et al. "Obesity and foot muscle strength are associated with high

dynamic plantar pressure during running." The Foot 44 (2020): 101683.

Naderi, Aynollah, Maarten H. Moen, and Hans Degens. "Is high soleus muscle activity

during the stance phase of the running cycle a potential risk factor for the development of

medial tibial stress syndrome? A prospective study." Journal of sports sciences 38.20

(2020): 2350-2358.

Naderi, Aynollah, et al. "Predictors of running-related injury among recreational runners: a

prospective cohort study of the role of perfectionism, mental toughness, and passion in

running." Sports health 16.6 (2024): 1038-1049.

Naderi, Aynollah, et al. "Effects of integrating lower-leg exercises into a multimodal

therapeutic approach on medial tibial stress syndrome management among recreational

runners: a randomized controlled study." Orthopaedic journal of sports medicine 13.2

(2025): 23259671241311849.

Naderi, Aynollah, Ali Yasemi, and Seyed Hamed Mousavi. "Effectiveness of an online multi-

component exercise-based injury prevention program on running-related injury risk in

recreational runners: a randomized controlled trial." Research in Sports Medicine 34.2

(2026): 217-237.

Integrating these references would enhance the theoretical depth, clinical relevance, and

global applicability of your manuscript.
