# Supplementary material for: Injury risk and running habits in parents: Does stroller use make a difference?
Source: PLoS One. 2026 Aug 12;21(8):e0354371. doi: 10.1371/journal.pone.0354371 (PMC13465853; doi:10.1371/journal.pone.0354371)
Supplement: Peer review history

# Peer review: Decision Letter 0

**DOI:** 10.1371/journal.pone.0354371.r001

**Authors:** Martin E. Matsumura

15 Apr 2026

PONE-D-26-09901

Injury risk and running habits in parents: Does stroller use make a difference?

PLOS One

Dear Dr. Altman-Singles,

Thank you for submitting your manuscript to PLOS ONE. After careful consideration, we feel that it has merit but does not fully meet PLOS ONE’s publication criteria as it currently stands. Therefore, we invite you to submit a revised version of the manuscript that addresses the points raised during the review process.

Please submit your revised manuscript by May 30 2026 11:59PM. If you will need more time than this to complete your revisions, please reply to this message or contact the journal office at plosone@plos.org. When you're ready to submit your revision, log on to https://www.editorialmanager.com/pone/ and select the 'Submissions Needing Revision' folder to locate your manuscript file.

Please include the following items when submitting your revised manuscript:

- A letter that responds to each point raised by the academic editor and reviewer(s). You should upload this letter as a separate file labeled 'Response to Reviewers'.
- A marked-up copy of your manuscript that highlights changes made to the original version. You should upload this as a separate file labeled 'Revised Manuscript with Track Changes'.
- An unmarked version of your revised paper without tracked changes. You should upload this as a separate file labeled 'Manuscript'.

If you would like to make changes to your financial disclosure, please include your updated statement in your cover letter. Guidelines for resubmitting your figure files are available below the reviewer comments at the end of this letter.

If applicable, we recommend that you deposit your laboratory protocols in protocols.io to enhance the reproducibility of your results. Protocols.io assigns your protocol its own identifier (DOI) so that it can be cited independently in the future. For instructions see: https://journals.plos.org/plosone/s/submission-guidelines#loc-laboratory-protocols. Additionally, PLOS ONE offers an option for publishing peer-reviewed Lab Protocol articles, which describe protocols hosted on protocols.io. Read more information on sharing protocols at https://plos.org/protocols?utm_medium=editorial-email&utm_source=authorletters&utm_campaign=protocols.

As the corresponding author, your ORCID iD is verified in the submission system and will appear in the published article. PLOS supports the use of ORCID, and we encourage all coauthors to register for an ORCID iD and use it as well. Please encourage your coauthors to verify their ORCID iD within the submission system before final acceptance, as unverified ORCID iDs will not appear in the published article. *Only*  the individual author can complete the verification step; PLOS staff *cannot*  verify ORCID iDs on behalf of authors.

We look forward to receiving your revised manuscript.

Kind regards,

Martin E. Matsumura, MD

Academic Editor

PLOS One

Journal Requirements:

When submitting your revision, we need you to address these additional requirements.

1. Please ensure that your manuscript meets PLOS ONE's style requirements, including those for file naming. The PLOS ONE style templates can be found at

https://journals.plos.org/plosone/s/file?id=wjVg/PLOSOne_formatting_sample_main_body.pdf and

https://journals.plos.org/plosone/s/file?id=ba62/PLOSOne_formatting_sample_title_authors_affiliations.pdf

2. When completing the data availability statement of the submission form, you indicated that you will make your data available on acceptance. We strongly recommend all authors decide on a data sharing plan before acceptance, as the process can be lengthy and hold up publication timelines. Please note that, though access restrictions are acceptable now, your entire data will need to be made freely accessible if your manuscript is accepted for publication. This policy applies to all data except where public deposition would breach compliance with the protocol approved by your research ethics board. If you are unable to adhere to our open data policy, please kindly revise your statement to explain your reasoning and we will seek the editor's input on an exemption. Please be assured that, once you have provided your new statement, the assessment of your exemption will not hold up the peer review process.

3. If the reviewer comments include a recommendation to cite specific previously published works, please review and evaluate these publications to determine whether they are relevant and should be cited. There is no requirement to cite these works unless the editor has indicated otherwise.

Additional Editor Comments:

A major concern is that the conclusions are not fully supported by the data. The authors need to revise the manuscript to better align conclusions with data:

1. Figs 3 +4: The authors describe the data as demonstrating "a trend toward significance." While this is techinically correct, I would prefer the authors not support this data with a non-significant p-value (p<0.1). A better place to discuss the "trend toward significance" and perhaps discuss the reason for this (likely sample size was underpowered to see a difference) would be in the discussion

2. The conclusion "stroller running is generally safe and accessible form of exercise for parent runners, with lower injury incidence observed compared to non-stroller runners," needs to be revised in light of point 1.

3. As a reviewer pointed out- the authors need to revise the discussion around incidence of plantar fasciitis. The text reads as though there were significant differences in this injury across groups, which is not supported by the data- I think the point is that the overall cohort had a notable high incidence of plantar fasciitis- is there evidence for higher risk of this injury among pregnant/early postpartum women?

[Note: HTML markup is below. Please do not edit.]

Reviewers' comments:

Reviewer's Responses to Questions

**Comments to the Author**

1. Is the manuscript technically sound, and do the data support the conclusions?

The manuscript must describe a technically sound piece of scientific research with data that supports the conclusions. Experiments must have been conducted rigorously, with appropriate controls, replication, and sample sizes. The conclusions must be drawn appropriately based on the data presented.

Reviewer #1: Yes

Reviewer #2: Partly

**********

2. Has the statistical analysis been performed appropriately and rigorously?

Reviewer #1: No

Reviewer #2: Yes

**********

3. Have the authors made all data underlying the findings in their manuscript fully available?

The PLOS Data policy requires authors to make all data underlying the findings described in their manuscript fully available without restriction, with rare exception (please refer to the Data Availability Statement in the manuscript PDF file). The data should be provided as part of the manuscript or its supporting information, or deposited to a public repository. For example, in addition to summary statistics, the data points behind means, medians and variance measures should be available. If there are restrictions on publicly sharing data—e.g. participant privacy or use of data from a third party—those must be specified.

Reviewer #1: No

Reviewer #2: Yes

**********

4. Is the manuscript presented in an intelligible fashion and written in standard English?

PLOS ONE does not copyedit accepted manuscripts, so the language in submitted articles must be clear, correct, and unambiguous. Any typographical or grammatical errors should be corrected at revision, so please note any specific errors here.

Reviewer #1: Yes

Reviewer #2: Yes

**********

5. Review Comments to the Author

Please use the space provided to explain your answers to the questions above. You may also include additional comments for the author, including concerns about dual publication, research ethics, or publication ethics. (Please upload your review as an attachment if it exceeds 20,000 characters)

Reviewer #1: Thank you for inviting me to review this interesting paper. The topic is very novel and important and is worthwhile research. I would like to commend the authors for researching in this important area. I believe the authors could make the abstract clearer to readers that this was solely a cross-sectional survey and not a longitudinal study as is somewhat implied in my opinion. The methods require improved clarity, the specific variables queried to runners is unclear and impacts the understanding of the results section. Also was a sample size calculation completed?

Please see information below that I hope the authors find useful.

Abstract:

1. Lines 36-38: Injury type and distribution were similar between groups, although there was a higher incidence of plantar fasciitis reported among both stroller and non-stroller runners.” – not sure why this is led in as an although, if it was high in both it should be “and” instead perhaps?

2. From reading later in the paper, this is a cross-sectional survey. This is not clear in this abstract and it implies that it is a 1.5 year longitudinal study.

3. The number of participants should be reported in the abstract.

4. The comparison between injury rate per 1,000 miles should clarify that it includes those with children aged 0-3 years as that is not clear

Introduction:

1. Lines 56-60: This sentence is quite hard to read and I am not sure what it adds really beyond the specific injuries noted earlier in the introduction section. I would consider removing this. I think solely from “Since about….” Is sufficient here.

2. Lines 129-131: “shown that decrease in..” – add an “a” there a decrease in.

Methods

1. It is slightly confusing to me that the start of the paragraph 1 in the methods section says it is exempt from ethics review but then later discusses what was approved?

2. The abstract implies that they were tracked for 1.5 years – whereas in reality this is a once off cross-sectional survey right? This is very unclear to the reader.

3. I am also very unclear about the period they are being asked about in the study. So to be eligible they had to have been a parent to children aged between 0-3 years within the last 20 years. So am I correct that this study is assessing people’s injuries just over a certain amount of time during that period? Or since then? The methods section is really lacking clarity on for what period the participants are reporting related to their injuries etc.

4. Was a sample size calculation completed for this study? Or a power calculation completed after the study was completed?

5. I am unable to comment on the statistical analysis utilised as I am not clear what data was exactly collected from the methods section. So while in the form I ticked no, I am not saying the data is incorrect, I just was unable this aspect based on the information above.

Results

1. Did you consider the impact of gender in the different groups?

2. Lines 258-260: I don’t think there is a need to consider “trending towards significance” – it was 0.07 and so was non-significant and should be stated as such.

3. Lines 276-279: This can be streamlined into one sentence.

4. Lines 276-290: This is a very long detailed paragraph that doesn’t really add much beyond what is outlined in a figure. This needs to be condensed.

5. What were the 95% CI for both groups for injury rates per 1,000 miles? That should be reported. Also solely 0-3 years was compared, is this because only injury during this time period was asked was it? The lack of clarity in the methods is impacting understanding of the results in my opinion and this should be rectified.

Discussion

1. Lines 269-370. Did you query sex or gender in the survey? The terms are being used interchangeably here and should align with what was specifically asked in the survey.

2. Lines 380-386. This is a typical issue with surveys. It should be part of the limitations section and shortened to one/two lines and condensed.

Note: There is a query on the review submission regarding the data being fully available. The statement in this document says that it will be published once accepted but I do not have access to it currently.

Reviewer #2: Thank you for submitting this timely and clinically relevant manuscript examining injury risk among parent runners who use strollers—a notably underexplored population in sports medicine. The study addresses an important public health question with generally clear writing and appropriate descriptive analyses. However, several methodological and reporting issues require revision before publication. The cross-sectional, self-reported survey design inherently limits causal inference, yet the discussion employs causal language (e.g., "confer a safeguarding effect") that overstates what the data can support; please revise to associative phrasing throughout. Key statistical reporting inconsistencies should be resolved, including the discrepancy between p=0.05 in the text versus "trend (p<0.1)" in Figure 4, inconsistent error bar metrics (SE vs. SD), and the unexplained "79% difference" claim. The operational definition of "overuse injury" requires clarification, and the absence of effect sizes, confidence intervals, and multivariable adjustment for potential confounders (training intensity, prior injury, footwear) limits interpretability. Additionally, please explicitly state the cross-sectional design in the abstract and methods per STROBE guidelines, address the gender imbalance (90% female in stroller group) and its implications for generalizability, and clarify instrument validation for the researcher-developed survey. With these revisions to strengthen methodological transparency, statistical rigor, and interpretive caution, this manuscript has strong potential to make a valuable contribution to the literature on postpartum physical activity and injury prevention. Recommendation: Major Revision.

**********

6. PLOS authors have the option to publish the peer review history of their article (what does this mean?). If published, this will include your full peer review and any attached files.

If you choose “no”, your identity will remain anonymous but your review may still be made public.

**Do you want your identity to be public for this peer review?**  For information about this choice, including consent withdrawal, please see our Privacy Policy.

Reviewer #1: No

Reviewer #2: No

**********

[NOTE: If reviewer comments were submitted as an attachment file, they will be attached to this email and accessible via the submission site. Please log into your account, locate the manuscript record, and check for the action link "View Attachments". If this link does not appear, there are no attachment files.]

To ensure your figures meet our technical requirements, please review our figure guidelines: https://journals.plos.org/plosone/s/figures

You may also use PLOS’s free figure tool, NAAS, to help you prepare publication quality figures: https://journals.plos.org/plosone/s/figures#loc-tools-for-figure-preparation.

NAAS will assess whether your figures meet our technical requirements by comparing each figure against our figure specifications.

Attachment Submitted filename: Manuscript Number.pdf

# Author comment: Author response to Decision Letter 1

**DOI:** 10.1371/journal.pone.0354371.r002

29 May 2026

Thank you for your thorough review of this manuscript. Our responses to the editor and reviewers’ comments are shown in red text (which can be viewed in the thorough Response to Reviewer Comments in the attachments). We appreciate the time and effort that went into each of these comments and have made all of the requested changes possible given the data set collected.

Journal Requirements:

When submitting your revision, we need you to address these additional requirements.

1. Please ensure that your manuscript meets PLOS ONE's style requirements, including those for file naming. The PLOS ONE style templates can be found at

https://journals.plos.org/plosone/s/file?id=wjVg/PLOSOne_formatting_sample_main_body.pdf and

https://journals.plos.org/plosone/s/file?id=ba62/PLOSOne_formatting_sample_title_authors_affiliations.pdf

We have adapted the manuscript and figure naming format to meet the journal’s formatting requirements.

2. When completing the data availability statement of the submission form, you indicated that you will make your data available on acceptance. We strongly recommend all authors decide on a data sharing plan before acceptance, as the process can be lengthy and hold up publication timelines. Please note that, though access restrictions are acceptable now, your entire data will need to be made freely accessible if your manuscript is accepted for publication. This policy applies to all data except where public deposition would breach compliance with the protocol approved by your research ethics board. If you are unable to adhere to our open data policy, please kindly revise your statement to explain your reasoning and we will seek the editor's input on an exemption. Please be assured that, once you have provided your new statement, the assessment of your exemption will not hold up the peer review process.

The data are now available at https://osf.io/et7nz/.

3. If the reviewer comments include a recommendation to cite specific previously published works, please review and evaluate these publications to determine whether they are relevant and should be cited. There is no requirement to cite these works unless the editor has indicated otherwise.

Additional citations have been added to reflect the editor and reviewers’ suggestions.

Additional Editor Comments:

A major concern is that the conclusions are not fully supported by the data. The authors need to revise the manuscript to better align conclusions with data:

1. Figs 3 +4: The authors describe the data as demonstrating "a trend toward significance." While this is techinically correct, I would prefer the authors not support this data with a non-significant p-value (p<0.1). A better place to discuss the "trend toward significance" and perhaps discuss the reason for this (likely sample size was underpowered to see a difference) would be in the discussion.

Thank you for this comment. We have removed the # indicators from the graphs and captions, and stated in the text that these results did not meet the criteria for statistical significance. We have included effect size estimates instead to demonstrate the meaning of the data presented.

2. The conclusion "stroller running is generally safe and accessible form of exercise for parent runners, with lower injury incidence observed compared to non-stroller runners," needs to be revised in light of point 1.

We see your point here; however, even the lack of statistically significant findings supports that there is no increase in running injury risk when running with a stroller. We’ve added text to clarify that the injury rates between groups were not different, indicating that running with a stroller is safe in the text to ensure the full message is communicated.

3. As a reviewer pointed out- the authors need to revise the discussion around incidence of plantar fasciitis. The text reads as though there were significant differences in this injury across groups, which is not supported by the data- I think the point is that the overall cohort had a notable high incidence of plantar fasciitis- is there evidence for higher risk of this injury among pregnant/early postpartum women?

There is some evidence that suggests incidence of plantar fasciitis may be affected by pregnancy (Foraldy et al., 2024). Whether that is due to hormonal fluctuations or weight gain is unclear, however, some evidence exists to suggest that plantar fascia thickness increases (Foraldy et al., 2024) and ligaments experience more laxity (Chu et al., 2019) during pregnancy. These morphological changes persist even after pregnancy, and thus, may in part cause this increase in plantar fasciitis observed in this population. References and text have been added to the manuscript to reflect this and clarify the concept.

5. Review Comments to the Author

Please use the space provided to explain your answers to the questions above. You may also include additional comments for the author, including concerns about dual publication, research ethics, or publication ethics. (Please upload your review as an attachment if it exceeds 20,000 characters)

Reviewer #1: Thank you for inviting me to review this interesting paper. The topic is very novel and important and is worthwhile research. I would like to commend the authors for researching in this important area. I believe the authors could make the abstract clearer to readers that this was solely a cross-sectional survey and not a longitudinal study as is somewhat implied in my opinion. The methods require improved clarity, the specific variables queried to runners is unclear and impacts the understanding of the results section. Also was a sample size calculation completed?

We thank the reviewers for this thorough review of the manuscript. The changes mentioned have been addressed here and in the comments below individually, with associated changes in the manuscript. We believe these changes to clarify the study design have helped to strengthen the manuscript.

Please see information below that I hope the authors find useful.

Abstract:

1. Lines 36-38: Injury type and distribution were similar between groups, although there was a higher incidence of plantar fasciitis reported among both stroller and non-stroller runners.” – not sure why this is led in as an although, if it was high in both it should be “and” instead perhaps?

We agreed and separated these two sentences to improve clarity and removed the “although” to prevent confusion.

2. From reading later in the paper, this is a cross-sectional survey. This is not clear in this abstract and it implies that it is a 1.5 year longitudinal study.

That is true. We have added that the study was a cross-sectional design in the abstract and clarified the language to ensure it is clear that subjects were recruited over a 1.5 year period, and not tracked over this period.

3. The number of participants should be reported in the abstract.

The group participant numbers have been added in the abstract.

4. The comparison between injury rate per 1,000 miles should clarify that it includes those with children aged 0-3 years as that is not clear

Text was added to the abstract to clarify that injuries were assessed from the first three years of parenting.

Introduction:

1. Lines 56-60: This sentence is quite hard to read and I am not sure what it adds really beyond the specific injuries noted earlier in the introduction section. I would consider removing this. I think solely from “Since about….” Is sufficient here.

The sentence in question was deleted and edits were made to the surrounding sentence to adjust the flow and clarify the injury breakdown.

2. Lines 129-131: “shown that decrease in..” – add an “a” there a decrease in.

Good catch, we added the “a” in this sentence.

Methods

1. It is slightly confusing to me that the start of the paragraph 1 in the methods section says it is exempt from ethics review but then later discusses what was approved?

Our apologies for the lack of clarity here; this was text we were required to add due to journal guidelines, and it doesn’t really make sense with an exempt study. I added that the consent method was approved “as exempt” from the institutional review board in the final sentence to ensure the statements are not contradictory.

2. The abstract implies that they were tracked for 1.5 years – whereas in reality this is a once off cross-sectional survey right? This is very unclear to the reader.

Clarifying text was added to the first paragraph of the methods section and the abstract to indicate that this was a cross-sectional study.

3. I am also very unclear about the period they are being asked about in the study. So to be eligible they had to have been a parent to children aged between 0-3 years within the last 20 years. So am I correct that this study is assessing people’s injuries just over a certain amount of time during that period? Or since then? The methods section is really lacking clarity on for what period the participants are reporting related to their injuries etc.

A sentence was added to clarify that runners were asked about their injuries during the period of time that is most commonly associated with stroller running, when their children were between 0 and 3 years of age.

4. Was a sample size calculation completed for this study? Or a power calculation completed after the study was completed?

An a priori power analysis was not completed at the onset of the study due to a lack of understanding of what we might find. Post hoc power analysis revealed that we were sufficiently powered in our Chi Squared calculation of the proportion of injured vs. healthy runners in each group, but may not have been sufficiently powered to observe a difference in injuries per 1000 miles run. We have made modifications to the text to reflect this.

5. I am unable to comment on the statistical analysis utilised as I am not clear what data was exactly collected from the methods section. So while in the form I ticked no, I am not saying the data is incorrect, I just was unable this aspect based on the information above.

Text was added to clarify the statistical methods used, and the data were provided via Open Science Framework https://osf.io/et7nz/.

Results

1. Did you consider the impact of gender in the different groups?

We considered the potential impact of gender in our analyses; however, both groups were predominantly composed of female runners, and the relatively small number of male participants limited meaningful gender-based comparisons. We assessed that the higher participation of female runners may itself be a notable finding, potentially reflecting that mothers within the parent runner population are more likely to identify as parents who run and participate in stroller running with their children, as they are more commonly the primary caregivers.

2. Lines 258-260: I don’t think there is a need to consider “trending towards significance” – it was 0.07 and so was non-significant and should be stated as such.

You are right in pointing this out. We have removed the phrase “trending towards significance” and revised the statement to appropriately report this result as not statistically significant. We have also added the calculated effect size to demonstrate a medium effect.

3. Lines 276-279: This can be streamlined into one sentence.

We have streamlined the text into a single sentence, improving conciseness.

4. Lines 276-290: This is a very long detailed paragraph that doesn’t really add much beyond what is outlined in a figure. This needs to be condensed.

We have condensed this paragraph to eliminate redundancy, and eliminated redundant information that can be viewed in Figure 5.

5. What were the 95% CI for both groups for injury rates per 1,000 miles? That should be reported. Also solely 0-3 years was compared, is this because only injury during this time period was asked was it? The lack of clarity in the methods is impacting understanding of the results in my opinion and this should be rectified.

The 95% confidence intervals for the stroller group were [0.09, 0.28] and the control group [0.13, 0.55]. This data has been added to the results for clarity. Additional text was added to the methods regarding the period of time requested.

Discussion

1. Lines 269-370. Did you query sex or gender in the survey? The terms are being used interchangeably here and should align with what was specifically asked in the survey.

Sex was queried from participants, and this term was used throughout the manuscript. “Gender bias” was used to describe the imbalance in recruitment of males vs. females, and this sentence has been corrected for clarity to avoid confusion between sex and gender.

2. Lines 380-386. This is a typical issue with surveys. It should be part of the limitations section and shortened to one/two lines and condensed.

We have moved the text to the limitations section and condensed it to two lines.

Note: There is a query on the review submission regarding the data being fully available. The statement in this document says that it will be published once accepted but I do not have access to it currently.

The data has been made available through Open Science Framework for the next round of review. It can be found at https://osf.io/et7nz/.

Reviewer #2: Thank you for submitting this timely and clinically relevant manuscript examining injury risk among parent runners who use strollers—a notably underexplored population in sports medicine. The study addresses an important public health question with generally clear writing and appropriate descriptive analyses. However, several methodological and reporting issues require revision before publication.

Thank you for your review of our work. We have attempted to address the methodological and reporting issues below. For clarity in our response, we separated out the reviewer’s comments into each topic and responded underneath.

The cross-sectional, self-reported survey design inherently limits causal inference, yet the discussion employs causal language (e.g., "confer a safeguarding effect") that overstates what the data can support; please revise to associative phrasing throughout.

We have revised the manuscript throughout, especially the discussion section, to remove causal language and replaced it with appropriate associative phrasing to better reflect the limitations of our study design and avoid overstatement of the data provided.

Key statistical reporting inconsistencies should be resolved, including the discrepancy between p=0.05 in the text versus "trend (p<0.1)" in Figure 4

The trend toward significance text has been removed throughout the manuscript based on the reviewers’ suggestions. The p-value of 0.05 is still included in the text along with an effect size calculation, demonstrating a small effect size.

inconsistent error bar metrics (SE vs. SD),

We’ve made changes for consistency, making all variation representations standard error rather than standard deviation (Table 1, Figure 2, and Figure 4).

and the unexplained "79% difference" claim.

The authors thank the reviewers for catching this. We retraced our steps and clarified the percentage of interest in the results first, then repeated the reduction in injury rate in the discussion.

The operational definition of "overuse injury" requires clarification

An operational definition of overuse injury has been added to the methods section indicating that any self-reported injury was considered for this study without consideration to clinical diagnosis, pain, duration, or impact on training. This is also discussed as a limitation in the discussion.

the absence of effect sizes, confidence intervals

Effect sizes and confidence intervals have been added throughout the text wherever appropriate.

multivariable adjustment for potential confounders (training intensity, prior injury, footwear) limits interpretability.

We have clarified in the Limitations section in our discussion that unmeasured confounding variables may have influenced the observed group differences.

Additionally, please explicitly state the cross-sectional design in the abstract and methods per STROBE guidelines

We have revised the abstract and methods to explicitly

Attachment Submitted filename: Injury Paper Reviewer Comments 4-15-2026.docx

# Peer review: Decision Letter 1

**DOI:** 10.1371/journal.pone.0354371.r003

**Authors:** Martin E. Matsumura

8 Jul 2026

Injury risk and running habits in parents: Does stroller use make a difference?

PONE-D-26-09901R1

Dear Dr. Altman-Singles,

We’re pleased to inform you that your manuscript has been judged scientifically suitable for publication and will be formally accepted for publication once it meets all outstanding technical requirements.

Within one week, you’ll receive an e-mail detailing the required amendments. When these have been addressed, you’ll receive a formal acceptance letter and your manuscript will be scheduled for publication.

An invoice will be generated when your article is formally accepted. Please note, if your institution has a publishing partnership with PLOS and your article meets the relevant criteria, all or part of your publication costs will be covered. Please make sure your user information is up-to-date by logging into Editorial Manager at Editorial Manager® and clicking the ‘Update My Information' link at the top of the page. For questions related to billing, please contact billing support.

If your institution or institutions have a press office, please notify them about your upcoming paper to help maximize its impact. If they’ll be preparing press materials, please inform our press team as soon as possible -- no later than 48 hours after receiving the formal acceptance. Your manuscript will remain under strict press embargo until 2 pm Eastern Time on the date of publication. For more information, please contact onepress@plos.org.

Kind regards,

Martin E. Matsumura, MD

Academic Editor

PLOS One

Additional Editor Comments (optional):

Reviewers' comments:

Reviewer's Responses to Questions

**Comments to the Author**

1. If the authors have adequately addressed your comments raised in a previous round of review and you feel that this manuscript is now acceptable for publication, you may indicate that here to bypass the “Comments to the Author” section, enter your conflict of interest statement in the “Confidential to Editor” section, and submit your "Accept" recommendation.

Reviewer #1: All comments have been addressed

**********

2. Is the manuscript technically sound, and do the data support the conclusions?

The manuscript must describe a technically sound piece of scientific research with data that supports the conclusions. Experiments must have been conducted rigorously, with appropriate controls, replication, and sample sizes. The conclusions must be drawn appropriately based on the data presented.

Reviewer #1: Yes

**********

3. Has the statistical analysis been performed appropriately and rigorously?

Reviewer #1: Yes

**********

4. Have the authors made all data underlying the findings in their manuscript fully available?

The PLOS Data policy requires authors to make all data underlying the findings described in their manuscript fully available without restriction, with rare exception (please refer to the Data Availability Statement in the manuscript PDF file). The data should be provided as part of the manuscript or its supporting information, or deposited to a public repository. For example, in addition to summary statistics, the data points behind means, medians and variance measures should be available. If there are restrictions on publicly sharing data—e.g. participant privacy or use of data from a third party—those must be specified.

Reviewer #1: Yes

**********

5. Is the manuscript presented in an intelligible fashion and written in standard English?

PLOS ONE does not copyedit accepted manuscripts, so the language in submitted articles must be clear, correct, and unambiguous. Any typographical or grammatical errors should be corrected at revision, so please note any specific errors here.

Reviewer #1: Yes

**********

6. Review Comments to the Author

Please use the space provided to explain your answers to the questions above. You may also include additional comments for the author, including concerns about dual publication, research ethics, or publication ethics. (Please upload your review as an attachment if it exceeds 20,000 characters)

Reviewer #1: Thank you to the authors for completing revisions. I only have one small suggestion for them to consider:

1. Remove the 1.5 year period – it is adding confusion and isn’t needed in the abstract in my opinion.

**********

7. PLOS authors have the option to publish the peer review history of their article (what does this mean?). If published, this will include your full peer review and any attached files.

If you choose “no”, your identity will remain anonymous but your review may still be made public.

**Do you want your identity to be public for this peer review?**  For information about this choice, including consent withdrawal, please see our Privacy Policy.

Reviewer #1: No

**********

# Editor report: Acceptance letter

**DOI:** 10.1371/journal.pone.0354371.r004

**Authors:** Martin E. Matsumura

PONE-D-26-09901R1

PLOS One

Dear Dr. Altman-Singles,

I'm pleased to inform you that your manuscript has been deemed suitable for publication in PLOS One. Congratulations! Your manuscript is now being handed over to our production team.

At this stage, our production department will prepare your paper for publication. This includes ensuring the following:

* All references, tables, and figures are properly cited

* All relevant supporting information is included in the manuscript submission,

* There are no issues that prevent the paper from being properly typeset

You will receive further instructions from the production team, including instructions on how to review your proof when it is ready. Please keep in mind that we are working through a large volume of accepted articles, so please give us a few days to review your paper and let you know the next and final steps.

Lastly, if your institution or institutions have a press office, please let them know about your upcoming paper now to help maximize its impact. If they'll be preparing press materials, please inform our press team within the next 48 hours. Your manuscript will remain under strict press embargo until 2 pm Eastern Time on the date of publication. For more information, please contact onepress@plos.org.

You will receive an invoice from PLOS for your publication fee after your manuscript has reached the completed accept phase. If you receive an email requesting payment before acceptance or for any other service, this may be a phishing scheme. Learn how to identify phishing emails and protect your accounts at https://explore.plos.org/phishing.

If we can help with anything else, please email us at customercare@plos.org.

Thank you for submitting your work to PLOS ONE and supporting open access.

Kind regards,

PLOS ONE Editorial Office Staff

on behalf of

Dr. Martin E. Matsumura

Academic Editor

PLOS One
